# Vitamin D status and blood pressure in children and adolescents: a systematic review of observational studies

**DOI:** 10.1186/s13643-021-01584-x

**Published:** 2021-02-22

**Authors:** Myriam Abboud, Fatme Al Anouti, Dimitrios Papandreou, Rana Rizk, Nadine Mahboub, Suzan Haidar

**Affiliations:** 1 Department of Health, College of Natural and Health Sciences, Zayed University, Dubai, United Arab Emirates; 2 Institut National de Santé Publique, d’Épidémiologie Clinique et de Toxicologie (INSPECT-Lb), Beirut, Lebanon; 3 Lebanese International University, Beirut, Lebanon; 4 Maastricht University, Maastricht, The Netherlands

**Keywords:** Vitamin D, Blood pressure, Children, Adolescents, Systematic review

## Abstract

**Background:**

Childhood hypertension is a growing public health problem. Simultaneously, hypovitaminosis D is widespread in this population and could be associated with hypertension. This study systematically reviewed the literature on the relationship between vitamin D status and blood pressure (BP) in children and adolescents.

**Methods:**

Following the PRISMA guidelines, PUBMED, MEDLINE, CINAHL, EMBASE, Cochrane Library, and ClinicalTrials.gov and the gray literature without language or time restrictions were searched. We included observational studies, assessed their risk of bias, and extracted data on population characteristics, vitamin D status and BP measurements, and the association between the two variables. A narrative analysis of the studies was performed.

**Results:**

In total, 85 studies were included. Prospective cohort studies showed no association between vitamin D and BP, and generally, they were flawed. Also, the majority of non-prospective cohort studies (cross-sectional, retrospective, case-control) did not report an association between vitamin D and BP. They were mostly flawed regarding BP measurement and adjusting to potential confounders.

**Conclusion:**

The results on the relationship between vitamin D status and BP in children and adolescents varied between the studies, and mainly pointed towards lack of association.

**Supplementary Information:**

The online version contains supplementary material available at 10.1186/s13643-021-01584-x.

## Background

Childhood hypertension is a serious public health burden, of considerable consequences [1]. In 2018, the global prevalence of childhood hypertension was estimated to be 4%, representing a substantial rise during the past two decades [2]. Between 2000 and 2015, the prevalence of hypertension showed a substantial increase among children aged 6 to 19 years [2]. This trend of increasing prevalence is expected to persist in the future, putting children at danger and further burdening health care systems [2]. Primary hypertension in childhood is commonly associated with cardiovascular risk factors, obesity, left ventricular hypertrophy, retinal vascular, and cognitive changes [3] and is associated with essential hypertension in adulthood [4, 5]. The increasing prevalence of hypertension is multifaceted, and its main driver is a higher body mass index (BMI) [2].

Simultaneously, hypovitaminosis D or low serum levels of hydroxyvitamin D (25(OH)D), is widespread in children and adolescents worldwide, even in countries which have plentiful sunlight all-year round [6–8]. The most common determinants of deficiency include limited exposure to sunlight, low dietary vitamin D intake, and sequestration in fat tissue especially among obese children and adolescents [9–12].

There is a general consensus that sufficient vitamin D levels during childhood promote skeletal growth and development. Yet, attention is being increasingly given to the extraskeletal benefits attained by having adequate vitamin D status [13]. Emerging evidence suggests that low serum vitamin D level is associated with poor health outcomes in the pediatric population, specifically obesity-related chronic health conditions, most notably hypertension [14, 15]. Obese youth with lower vitamin D levels have showed increased odds for hypertension, and this association remained significant even after adjusting for either BMI or total fat mass [16]. Moreover, an inverse association between 25(OH)D and systolic blood pressure (SBP) has been noted [17]. Several biologically plausible hypotheses support the beneficial effect of adequate vitamin D status on blood pressure (BP), including the role of vitamin D in improving endothelial function, decreasing proinflammatory cytokine levels, and regulating the renin-angiotension-aldosterone system [18–23].

To date, human interventional studies have failed to produce conclusive evidence pertaining to vitamin D as a potential antihypertensive supplement. A recent systematic review conducted by Abboud [24] revealed no benefit of vitamin D supplementation on decreasing SBP or diastolic blood pressure (DBP). This could be explained by the fact that other factors, rather than simply dietary intake, determine vitamin D status and its health implications. Vitamin D status can vary quite markedly in groups of people with apparently similar input level and is affected by calcium intake, some therapeutic agents, adiposity levels, and exercise [25]. Attaining adequate vitamin D status for preventing hypertension could thus be of public health relevance. Therefore, this study aims to systematically review the literature to decipher the relationship between vitamin D status and BP in children and adolescents.

## Methods

### Review design

This is a systematic review conducted according to the Preferred Reporting Items for Systematic Reviews and Meta-Analyses (PRISMA) statement [26] (see Additional file 1) and following a predefined protocol that was registered with the International Prospective Register of Systematic Reviews (PROSPERO) (CRD42020167550). Ethical approval was not required for this purpose.

The databases PUBMED, MEDLINE (Ovid), CINAHL (EBSCO), EMBASE (Ovid), the Cochrane Library, and http://www.ClinicalTrials.gov, were searched as well as the references of included articles, and previous reviews of vitamin D and BP in children and adolescents identified by the search. The search included observational studies reporting on the association between vitamin D status and BP (systolic, diastolic, or mean arterial pressure (MAP)) in children and adolescents. There were no language or time restrictions to eligible reports.

### Search strategy

Our search strategy included three key concepts: (1) vitamin D, (2) blood pressure, and (3) children and adolescents, and for each concept, we mapped Medical Subject Headings (MeSH) and keywords. The search included terms such as vitamin D, cholecalciferol, ergocalciferol, or calcidol, combined with BP or hypertension, and pediatric, child, adolescent, youth, or teenage. We did not apply time restrictions to the search, i.e., the databases were searched from their inception date through January 17, 2020. In addition, we conducted a search update on June 09, 2020. A medical information specialist validated the electronic search strategy (see Additional file 2). The references retrieved from scientific databases were managed using EndNote software, version X6.

### Study selection

The selection included prospective cohort, cross-sectional, case-control, or retrospective studies including children and adolescents as defined by the studies (e.g., aged less than 18 years). Observational studies evaluating the relationship between vitamin D status (e.g., 25(OH)D blood level) and BP were also considered. The outcomes of interest included the difference in measured BP readings, or the odds of increased BP, or the differences in the prevalence of hypertension between participants with suboptimal vitamin D status, e.g., deficiency, and those with adequate levels of vitamin D. The outcomes of interest also included the correlation between vitamin D levels and BP levels, and the difference in vitamin D levels across groups of BP (e.g., normotensive vs. hypertensive). Mixed studies (including children, adolescents, and adults) were included if there was a subgroup analysis for children and/or adolescents only.

This review excluded studies evaluating the effect of vitamin D supplementation on BP reduction as well as studies investigating the association between hypervitaminosis D and BP. Also excluded were studies conducted on adult participants as defined by the studies (e.g., aged 18 years and above), pregnant women, neonates (aged 0 to 30 days), and infants (aged 1 month to 2 years), as classified by the World Health Organization, participants with diseases affecting vitamin D metabolism (e.g., chronic kidney disease, dialysis, liver disease, parathyroid abnormality, and vitamin D-dependent rickets types 1 and 2), participants taking medications known to interfere with vitamin D metabolism (e.g., phenytoin, phenobarbital, carbamazepine, and rifampin).

Screening of titles and/or abstracts retrieved by the search was done in duplicate on EndNote software, version X6, and eligible studies were identified. Two pairs of authors (M.A. and R.R.; N.M. and S.H.) then retrieved the full texts of these studies and assessed them for eligibility in duplicate. Disagreements were solved through discussion. A calibration exercise was conducted before study selection to ensure the validity of the process.

### Data extraction

Data from eligible studies were extracted in duplicate by two pairs of authors (M.A. and R.R.; N.M. and S.H.) on Microsoft Excel 2016, following a data extraction standard form. A calibration exercise was first conducted. Disagreements were resolved through discussion The following details were retained: characteristics of the study, details of the population included, the studied exposures and outcomes, and the main findings and adjustments to the analyses, as applicable. Serum 25(OH)D measures were converted to nanomoles per liter whenever it was reported as nanograms per milliliter, by multiplying by a factor of 2.496.

### Risk of bias assessment

After an initial calibration exercise, two pairs of authors (M.A. and R.R.; N.M. and S.H.) collaboratively assessed in duplicate the risk of bias of included studies, solving disagreements between them through discussion. We used a modified version of the Cochrane Risk of Bias tool [27] that is designed to assess to risk of bias of observational studies. Each potential source of bias was graded as low, high, or unclear risk. The criteria for judging a high risk of bias included failure to develop and apply appropriate eligibility criteria; flawed measurement of both exposure and outcome; failure to adequately control confounding; and incomplete follow-up (only for prospective cohort studies).

### Data synthesis

Separate narrative analyses of the findings of prospective cohort and non-prospective cohort studies were performed. Furthermore, we provided separate analyses for non-prospective studies based on the level of adjustment for confounding factors. We opted for this method of analysis because failure to take into account confounding factors decreases the quality of the evidence generated by the study [27].

## Results

### Search results

**Fig. 1:**
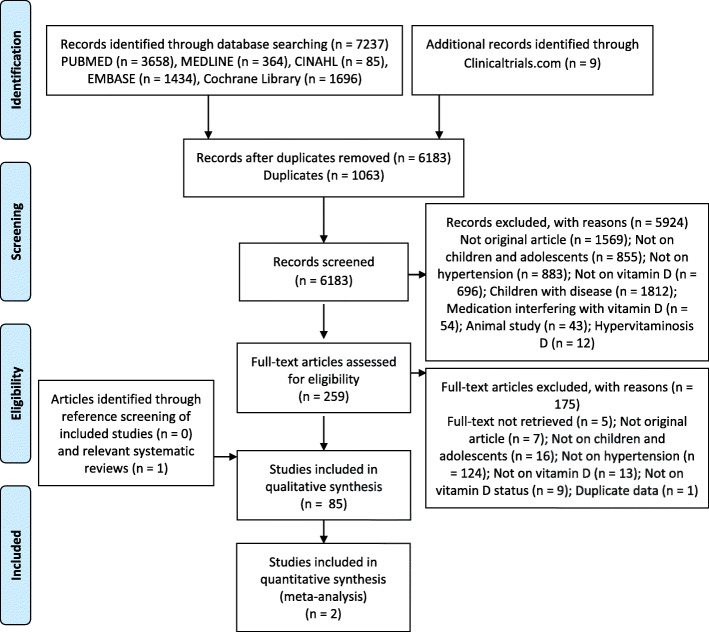
PRISMA diagram of study selection

The details of the search process are detailed in Fig. 1. A total of 85 studies [14–16, 28–108] were included in the systematic review.

### Prospective cohort studies

#### Characteristics of the studies

We identified three prospective cohort studies [60, 99, 102], which characteristics are detailed in Table 1. These studies were published between 2014 [102] and 2019 [99], and were conducted in Australia [60], United States of America (USA) [99], and England [102].

**Table 1:** Characteristics and results of prospective cohort studies

First author, year- country	Study population	Outcomes evaluated and evaluation method	Adjusted variables for statistical analysis	Key findings
Ke, 2018- Australia [60]	249 healthy Australian children recruited at age 8, 162 followed up at age 15 Mean BMI (SD) in kg/m^2^ at 8 years: 16.7(2.3) Mean BMI (SD) in kg/m^2^ at 15 years: 22.2 (3.8) % Male at age 8: 47.4%; at age 15: 54.3% Age in years: Range: 5.9–8.5; Mean (SD) at 8 years: 7.8 (0.6); at 15 years: 14.9 (0.2)	Serum 25(OH)D: Radioimmunoassay SBP and DBP: average of 3 readings by an automated BP monitor after a 5 min rest	Age; Date of blood draw; Sum of skinfolds; Socioeconomic status; Body fat	● At 8 years old: No association between 25(OH)D and log SBP (standardized β: 0.004; NS); and log DBP (standardized β: − 0.049; NS) ● At 15 years old: No association between 25(OH)D and log SBP (standardized β: − 0.06; NS); Significant inverse association between 25(OH)D and log DBP (standardized β: − 0.19; *p* = 0.015) ● No association between 25(OH)D at age 8 and BP at age 15 in boys [SBP (standardized β: − 0.18; *p* = 0.2); DBP (standardized β: -0.06; NS)] and girls [SBP (standardized β: 0.11; NS); DBP (standardized β: − 0.1; NS)]
Wang, 2019- Boson, USA [99]	775 black and other children from the original Boston Birth Cohort, enrolled from 2005 to 2012 and followed prospectively up to age 18 years at a medical center Mean BMI (SD) in kg/m^2^: NR % Male: 49.8% Age in years: Median (IQR) for last BP measurement: 10.7 (8.7–12.3)	Plasma 25(OH)D2 and 25(OH)D3: HPLC-tandem mass spectrometry assay SBP: validated automatic sphygmomanometer, on the right arm, in a sitting position with an appropriately sized cuff Elevated SBP: BP ≥ 75th p for age, gender, and height	Maternal age; Race; Education; Smoking; Parity; Hypertensive disorder; Diabetes mellitus; Pre-pregnancy obesity; Preterm birth; Birthweight; Season at birth and season at measurement; Breastfeeding; Child’s age at BP measurement; Sex; Current overweight or obesity None for vitamin D across SBP groups	● No association between low vitamin D status during early childhood and SBP at ages 3 to 5 years. Higher odds of elevated SBP at 6 to 18 years of age with low vitamin D status in early childhood, rendered not significant when adjusted for current weight status: OR for low vitamin D status during early childhood and SBP at 3–5 years [OR 0.96 (95%CI 0.62–1.49); *p* = 0.859] and 6*–*18 years [OR 1.56 (95%CI 1.00–2.44); *p* = 0.051] ● No difference in plasma 25(OH)D in childhood, according to Child Systolic BP p: 83.12 (28.2) nmol/L for < 75 p vs. 78.87 (24.71) nmol/L for ≥ 75 p; *p* = 0.050
Williams, 2014- Avon, Southwest England [102]	2470 participants of the Avon Longitudinal Study of Parents and Children at 15-year of follow-up Mean (SD) BMI: 21.5 (3.7) % Male: 49.5% Age in years: Range: 7–12; Mean: 15.4; Mean (SD) at 25(OH)D sampling: 10.0 (0.9)	25(OH)D2 and 25(OH)D3: NR SBP and DBP: NR	Age; Gender; Socioeconomic status; Childhood BMI; Follow-up BMI; Season-adjusted 25(OH)D3; PTH; Circulating calcium and phosphate	NS association between vitamin D measured during childhood (at mean age 7.4, 9.8, or 11.7 years) and BP at mean age 15.5 years ● Mean difference per doubling of 25(OH)D2 SBP: Mean difference: − 0.12 (95% CI − 0.61, 0.39); *p* = 0.65 DBP: Mean difference: − 0.10 (95% CI − 0.59, 0.37); *p* = 0.68 ● Mean difference per doubling of 25(OH)D3 SBP: Mean difference: 0.47 (95% CI − 0.45, 1.35); *p* = 0.29 DBP: Mean difference: − 0.06 (95% CI − 0.87, 0.75); *p* = 0.88 ● Mean difference between 25(OH)D 50–72 nmol/L vs. 25(OH)D ≥ 72 nmol/L SBP: Mean difference: 0.58 (95% CI − 0.41, 1.57) DBP: Mean difference: 0.65 (95% CI − 0.18, 1.48) ● Mean difference between 25(OH)D < 50 nmol/L vs. 25(OH)D ≥ 72 nmol/L SBP: Mean difference: − 0.60 (95% CI − 1.69, 0.49) DBP: Mean difference: 0.17 (95% CI − 0.75, 1.08)

*BMI* body mass index, *SD* standard deviation, *25(OH)D* 25-hydroxyvitamin D, *SBP* systolic blood pressure, *DBP* diastolic blood pressure, *BP* blood pressure, *Β* beta, *NS* not significant, *USA* United States of America, *NR* not reported, *IQR* interquartile range, *25(OH)D2* 25-hydroxyvitamin D2, *25(OH)D3* 25-hydroxyvitamin D3, *HPLC* high-performance liquid chromatography, *p* Percentile, *OR* odds ratio, *CI* confidence interval

#### Results of the studies

The findings from the prospective cohort studies are also presented in Table 1. Two [60, 102] out of three studies showed no association between vitamin D and BP (SBP and DBP), whereas one [99] showed an inverse association between low vitamin D status during early childhood and SBP a decade later, which was rendered not significant after adjustment for weight status.

In details, Ke et al. [60] examined 25(OH)D concentrations in an Australian cohort of 8-year-olds (*n* = 249) followed up at age 15 (*n* = 162) and explored associations between 25(OH)D with cardiovascular disease (CVD) risk factors, including BP, in these populations. On the cross-sectional analyses of 8- and 15-year-olds, SBP and DBP were not found to be significantly associated with 25(OH)D concentrations. Of interest, prospectively there was no association between 25(OH)D at age 8 and SBP nor DBP at age 15, both in boys and in girls. Similarly, Williams et al. [102] compared prospective associations of two analogs of childhood 25(OH)D (25(OH)D2 and 25(OH)D3) at ages 7–12 years with SBP and DBP measured in adolescence. The analyses were conducted on 2470 participants of the Avon Longitudinal Study of Parents and Children (ALSPAC)—a prospective birth cohort that recruited pregnant women in the former county of Avon, South West England. The results of this study showed that there were no associations between vitamin D measured during childhood with SBP and DBP at mean age of 15.5 years. Finally, among 775 children from the Children’s Health Study (CHS)—a prospective birth cohort study recruiting children from the original Boston Birth Cohort at Boston Medical Center, Wang et al. [99] investigated whether vitamin D status in early life can affect SBP a decade later and reported not association between the two variables at ages 3 to 5 years. Further, Wang et al. [99] reported higher odds of elevated SBP at age 6 to 18 years among children with lower vitamin D during early childhood; however, this result was rendered not significant when adjusted for current weight status. Among the three studies, only Wang et al. [99] studied vitamin D levels across SBP status groups (< 75^th^ percentile vs. ≥ 75^th^ percentile) during childhood and reported no association between the two variables (*p* = 0.05).

#### Assessment of risk of bias

**Fig. 2:**
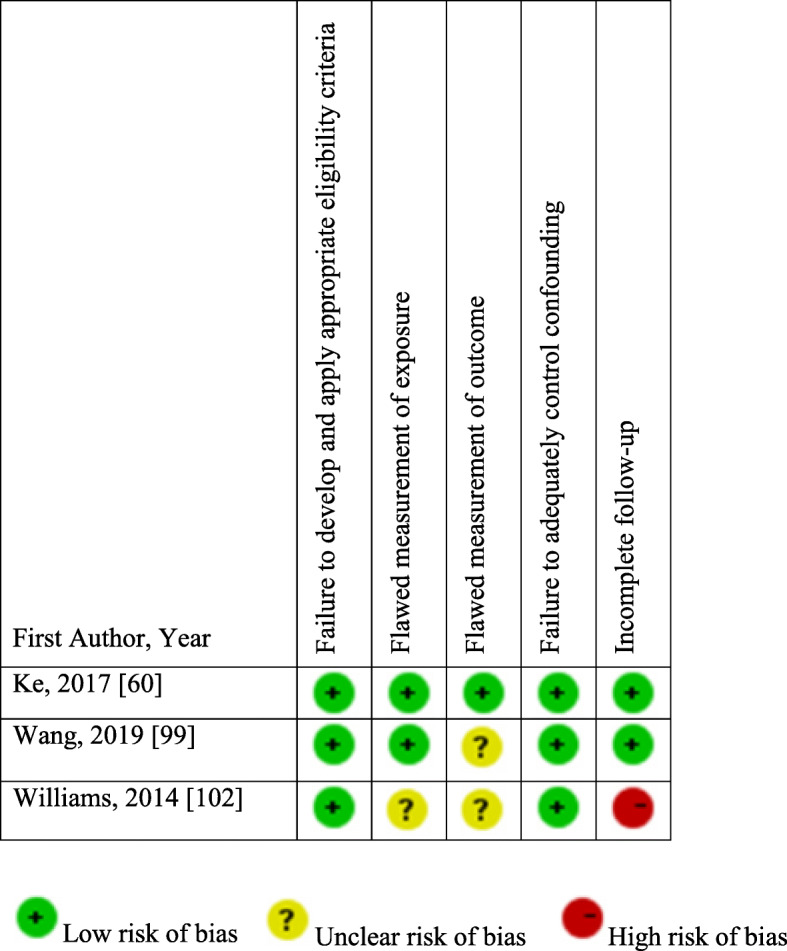
Risk of bias of prospective cohort studies

The assessment of risk of bias of prospective cohort studies is presented in Fig. 2. Developing and/or applying appropriate eligibility criteria were appropriate in the three studies. The assessment of vitamin D was adequate in Ke et al. [60] and Wang et al. [99], yet not reported in Williams et al. [102]. The assessment of BP was adequate in Ke et al. [60], yet, it was not reported in Williams et al. [102]. In Wang et al. [99], the number of BP measurements was not reported; thus, the risk of bias was unclear. The three studies extensively adjusted to potential confounders regarding the association between vitamin D and BP. Finally, there was no suspicion of differential loss to follow-up in Ke et al. [60] and Wang et al. [99]; however, in Williams et al. [102], there were numerous significant differences in the characteristics of included and excluded participants because of missing data on one or more variable, which might imply a high risk of bias.

### Non- prospective cohort studies (cross-sectional, retrospective and case-control studies)

#### Characteristics of the studies

Table 2 details the characteristics of non-prospective cohort studies. The included studies were published between 2007 [95] and 2020 [47, 57, 82, 92, 105]. The majority of the studies were conducted in the Americas (USA, *n* = 23 [15, 33, 34, 36, 37, 40, 43, 65, 66, 69, 75, 79–81, 84, 86, 91, 92, 95, 100, 103, 107]; Latin America, *n* = 9 [53–56, 74, 83, 96–98]; Canada, *n* = 1 [71]), followed by Asia (*n* = 33) [14, 28–32, 35, 38, 39, 44, 48–50, 57–59, 61–64, 67, 68, 70, 72, 73, 76–78, 88, 90, 105, 106, 109], Europe (*n* = 14) [42, 45–47, 51, 82, 85, 87, 89, 93, 94, 101, 104, 108], Australia (*n* = 1) [16], and Africa (*n* = 1) [52]. The majority of the studies were cross-sectional (*n* = 67) [14, 15, 28, 29, 31–38, 40–48, 50–59, 61, 62, 64, 65, 68, 69, 72–78, 80, 81, 83–93, 96–98, 100, 104–109], and there were seven retrospective studies [16, 39, 49, 66, 71, 95, 103], one case-control study [70]. We also identified four articles [30, 63, 82, 94] which included a cross-sectional baseline assessment from human interventional studies, and two from prospective cohort studies [67, 101] and one article [79] included data from both prospective cohort (baseline assessment) and cross-sectional studies. The sample size ranged from 22 [35] to 9757 [65] participants; the age of participants ranged from 1 to 21 years [65]. Five studies included females only [36, 37, 58, 63, 90], while one study [53] consisted of males only. Twelve studies [15, 61, 64, 65, 68, 75, 77–79, 91, 100, 105] included nationally representative samples. Twenty studies included obese/overweight participants [16, 33, 34, 37, 38, 41, 47, 49, 62, 69, 71, 84, 88, 89, 95, 96, 98, 103, 104, 107]; one study [43] included children with multiple, modifiable atherosclerosis-promoting risk factors; one [79] was conducted on youth with type 1 diabetes. Only one study [93] recruited children and adolescents with primary hypertension, and only one [35] included healthy children with vitamin D deficiency.

**Table 2:** Characteristics and results of non-prospective cohort studies

First author, year- country	Study population	Outcomes evaluated and evaluation method	Adjusted variables for statistical analysis	Key findings
**Cross-sectional**				
Al Daghri, 2010- Riyadh, Saudi Arabia [28]	118 children and adolescents, non-obese, without chronic disease, not taking vitamin D supplements, recruited from primary health care centers Mean BMI (SD) in kg/m^2^: 118 children and adolescents, non-obese, without chronic disease, not taking vitamin D supplements, recruited from primary health care centers Mean BMI (SD) in kg/m^2^: Boys: 19.8 (5.7); Girls: 18.9 (4.3) % Male: 44.9% Age in years: Range: 5–17; Mean(SD): Boys: 12.4 (3.7); Girls: 11.6 (3.7)	Serum 25(OH)D: ELISA SBP and DBP: average of 2 readings (not detailed)	None	Inverse correlation between vitamin D with SBP and DBP in the total sample and in girls, but not in boys
Al Daghri, 2015- Riyadh, Saudi Arabia [29]	259 children, healthy, not taking medications or supplements known to affect body weight, recruited from primary health care centers Mean BMI(SD) in kg/m^2^: Boys: 23.38 (6.23); Girls: 22.95 (4.97) % Male: 39.8% Age in years: Range: NR; Mean(SD): Boys 14.9 (1.6); Girls 14.8 (1.6)	Serum 25(OH)D: COBAS e-411 automated analyzer SBP and DBP: NR	None	● Inverse correlation between vitamin D with SBP in boys only ● Inverse correlation between vitamin D with DBP in the total sample
Al Daghri, 2015- Riyadh, Saudi Arabia [31]	2225 adolescents, healthy, without acute or chronic medical conditions, recruited from private and public schools Mean BMI(SD) in kg/m^2^: Boys: 22.9 (0.17); Girls: 23 (0.16) % Male: 53.3% Age in years: Range: 13–17; Mean(SD): Boys: 15.1 (0.06); Girls: 15.1 (0.06)	Serum 25(OH)D: COBAS e-411 automated analyzer SBP and DBP: average of 2 readings, 15 min apart, by a standardized mercury sphygmomanometer	None	Inverse correlation between vitamin D with SBP and DBP in boys only
Al Daghri, 2016- Riyadh, Saudi Arabia [30]	4183 children, without acute medical condition, with information collected from a biomarkers research program Mean BMI(SD) in kg/m^2^: Boys: 21.4 (5.1); Girls: 22 (4.8) % Male: 45.6% Age in years: Range: 12–18; Mean(SD): Boys: 14.3 (1.9); Girls: 14.3 (2.1)	Serum 25(OH)D: COBAS e-411 automated analyzer SBP and DBP: NR	None	Inverse correlation between vitamin D with SBP and DBP in boys only
Al Daghri, 2018- Riyadh, Saudi Arabia [32]	740 adolescents, without serious medical conditions, not taking medications or vitamin D supplements, recruited from primary schools Mean BMI(SD) in kg/m^2^: 21.9 (4.8) % Male: 33.1% Age in years: Range: 10–17; Mean(SD): All: 14.2 (1.6); Boys:14.1 (1.2); Girls: 14.3 (1.8)	Serum 25(OH)D: COBAS e-411 automated analyzer Vitamin D status groups: Deficient: 50–75 nmol/L; Insufficient: < 50 nmol/L; Sufficient: ≥ 75 nmol/L SBP and DBP: NR	None	● No difference in SBP across vitamin D status groups ● Lower DBP in vitamin D sufficiency
Al Saleh, 2013-Riyadh, Saudi Arabia [35]	22 children with vitamin D deficiency, free of chronic diseases, not taking vitamin D supplements, recruited from primary health care centers Mean BMI(SD) in kg/m^2^: NR % Male: 43.4% Age in years: Range: NR; Mean(SD): NR	Serum 1,25(OH)2D: ELISA SBP and DBP: average of 2 readings (not detailed)	Gender and BMI	Inverse association between vitamin D with SBP only
Alemzadeh, 2012-Wisconsin, USA [33]	133 Caucasian, Hispanic and African adolescents, obese (BMI > 95th p for age), medically stable, not taking medications or multivitamin supplement, recruited from an endocrine clinic Mean BMI(SD) in kg/m^2^: NR % Male: 41.4% Age in years: Range: 13.1–17.9; Mean(SD): 14.9 (1.4)	Serum 25(OH)D: radioimmunoassay Vitamin D status groups: Deficient: < 50 nmol/L; Sufficient: ≥ 50 nmol/L SBP and DBP: average of 2 readings, in sitting position	None	● No differences in SBP and DBP across vitamin D status groups ● No correlation between vitamin D with SBP and DBP
Alemzadeh, 2016-Wisconsin, USA [34]	152 Caucasian, Hispanic and African adolescents, obese (BMI > 95th p for age), without chronic medical conditions, not taking supplements, recruited from a children hospital Mean BMI(SD) in kg/m^2^: NR % Male: 42.8% Age in years: Range: 13.2-17.8; Mean(SD): 14.7 (1.3)	Serum 25(OH)D: radioimmunoassay Vitamin D status groups: Deficient: < 50 nmol/L; Insufficient: 50–74.9 nmol/L; Sufficient: ≥ 75 nmol/L SBP and DBP: average of 2 readings, in sitting position	None	No differences in SBP and DBP across vitamin D status groups
Ashraf, 2011- Birmingham, USA [37]	80 Caucasian American and African American post-menarchal adolescents, obese (BMI > 95th p for age and gender), without chronic disease, not taking supplements, recruited from weight management clinics Mean BMI(SD) in kg/m^2^: NR % Male: 0% Age in years: Range: NR; Mean(SD): African American: 14.3 (2.3), Caucasian American: 14.8 (2.3)	Serum 25(OH)D: liquid chromatography-tandem mass spectrometry SBP and DBP: automated BP cuff (not detailed)	BMI; Race	No correlation between vitamin D with SBP and DBP
Ashraf, 2014- Alabama, USA [36]	47 European American and African American post-menarchal adolescents, without medical conditions, not taking medications and supplements, recruited from weight management clinics Mean BMI(SD) in kg/m^2^: 23.3 (4.5) %Male: 0% Age in years: Range: 14-18; Mean(SD): 15.9 (1.4)	Serum 25(OH)D: liquid chromatography mass spectrometry Free 25(OH)D and bioavailable 25(OH)D: calculated using published formulas SBP ad DBP: average of 2 readings, after a 5-min rest, using the auscultatory method, in supine position	Age; Percent body fat; Race; Fasting insulin; Height	No correlation between vitamin D with SBP and DBP
Atabek, 2014- Konya, Turkey [38]	247 children and adolescents, obese (BMI > 95 p for age and gender), without chronic disease, not taking medications, recruited from an outpatient endocrinology and diabetes pediatric clinic Mean BMI(SD) in kg/m^2^: NR %Male: 47.3% Age in years: Range: 8–16; Mean(SD): 11.93 (2.77)	Serum 25(OH)D: mass spectrometry Vitamin D status groups: Deficient:< 50 nmol/L SBP and DBP: after a rest ≥ 10 min, using a standard mercury sphygmomanometer	None	● No differences in SBP and DBP across vitamin D status groups ● Inverse correlation between vitamin D with SBP only
Bacha, 2019- Texas, USA [40]	79 Hispanic, Black and White American adolescents, not engaging in a diet or physical activity program, without medical conditions, not taking medications and supplements, recruited through advertisement in the community and at a children’s hospital Normal weight:22.8%; overweight with normal glucose tolerance:38%; overweight with prediabetes:39.2% %Male: 43% Age in years: Range: NR; Mean(SD): 15.4 (0.2)	Total 25(OH)D: electrochemiluminescence assay SBP and DBP: average of 7 readings, taken 10 min apart, by an automated device	None	No differences in SBP and DBP across tertiles of vitamin D
Banzato, 2014- Verona, Italy [41]	32 Caucasian children, overweight and obese (definition: NR), free of chronic disease, not taking medications or supplements, recruited from a pediatric department Mean BMI(SD) in kg/m^2^: 30.08 (3.18) %Male: 65.6% Age in years: Range: 7–16; Mean(SD): 11.7(2.26)	25(OH)D: chemiluminescent method SBP and DBP: average of 3 readings, on the left arm over 30 min, in sitting position, using a mercury sphygmomanometer	None	No differences in SBP and DBP across tertiles of vitamin D
Cabral, 2016- Porto, Portugal [42]	514 adolescents from the EPITeen, recruited from public and private schools BMI ≥ 95 p for age and gender: 9.3% % Male: 47.5% Age in years: Range: 13; Mean(SD): NR	Serum 25(OH)D: chemiluminescence immunoassay SBP and DBP: average of 2 readings separated by ≥ 5 min, after a 10-min rest, by a mercury sphygmomanometer	BMI; Gender; Parental education; Physical activity; Season	● No differences in SBP and DBP across quartiles of vitamin D ● No difference in vitamin D level among normotensive and those with high BP
Cheraghi, 2012- Kansas, USA [43]	74 white and non-white children with multiple, modifiable atherosclerosis-promoting risk factors, recruited from a preventive cardiology clinic at a children's hospital BMI > 95 p for age and gender: 85% %Male: 44.6% Age in years: Range: NR; Mean(SD): 13.7 (3.1)	Serum 25(OH)D: NR Vitamin D status groups: Deficient: < 49.92 nmol/L; Sufficient: ≥ 49.92 nmol/L SBP: over the right arm, in sitting position, using a dinamap monitor	None	● No difference in SBP across vitamin D status groups ● No difference in vitamin D level among those with normal and high SBP
Choi, 2014- South Korea [44]	260 adolescents, free of diabetes, recruited from a rural high school Mean BMI(SD) in kg/m^2^: Boys:22.2 (3.2); Girls: 21.2 (2.5) %Male: 51.9% Age in years: Range: 15–16; Mean(SD): 15.9 (0.3)	Serum 25(OH)D: radioimmunoassay SBP and DBP: average of 2 readings, at rest, using a oscillometric device with appropriate cuff size	None	No correlation between vitamin D with SBP and DBP
De Moraes, 2014- European Countries [45]	1089 European adolescents from the HELENA study, recruited from different European countries Mean BMI(SD) in kg/m^2^: Boys:21.4; Girls: 21.3 %Male; 46.7% Age in years: Range: 12.5–17.5; Mean: 14.8	Plasma 25(OH)D: immunoassay ELISA SBP and DBP: lowest of 2 readings, taken 10 min apart, in sitting position, using an oscillometric monitor device	Contextual variables (seasonality; latitude of residence; school); Potential individual confounders (maternal education; age at menarche (in girls); BMI; waist circumference; physical activity; serum lipid concentrations); Biomarker serum concentrates (blood composition; iron status indicators; multiple vitamins including vitamin D)	No association between vitamin D with SBP and DBP
De piero Belmont, 2015-Spain [46]	314 children, free of disease, not taking medications and supplements, recruited from public schools Mean BMI(SD) in kg/m^2^: 19.4 (3.2) %Male: 50.3% Age in years: Range: 8–13; Mean (SD): 10.7 (1.0)	Plasma 25(OH)D: immunochemiluminescence SBP and DBP: average of 3 reading, taken 5 min apart using the right arm, in sitting position HTN status: Normal BP: SBP or DBP ≤ 90th p; Pre-HTN: SBP or DBP ≥ 90th p and ≤ 95 thp; High BP: SBP or DBP ≥ 95th p	For the OR: Age; gender	● Lower SBP and DBP with increasing tertiles of vitamin D ● Higher prevalence of HTN in the lowest tertile of vitamin D compared with the highest tertile ● Lower odds of elevated SBP or DBP with increasing tertiles of vitamin D
Dura-Trave, 2020- Pamplona, Spain [47]	236 Caucasian adolescents, with severe obesity (BMI *z*-score > 3.0 or BMI > 99th p), without illness, not taking medication, vitamin D, or calcium supplements Mean BMI *z*-score(SD): deficiency: 4.3 (1.1); insufficiency: 4.4 (0.9); sufficiency: 3.8 (0.6) %Male: 62.3% Age in years: Range: 10.2–15.8; Mean: 13.4	Plasma 25(OH)D: high-specific chemiluminescence immunoassay Vitamin D status group: Deficient: < 50 nmol/L; hypovitaminosis D: < 75 nmol/L SBP and DBP: lowest of 3 measurements, in the right arm, in supine position, using a digital BP monitor Arterial HTN: SBP and/or DBP ≥ 95th p for age, sex, and height, according to the American reference charts (SBP > 130 or DBP > 85 mmHg)	None	● Higher SBP and DBP with vitamin D deficiency ● Higher prevalence of arterial HTN with hypovitaminosis D ● Inverse association between vitamin D with SBP only
Ganji, 2011-USA [15]	5867 White, Black and Hispanic children and adolescents, recruited from the NHANES study (2001–2006) Mean BMI(SD) in kg/m^2^: 23.1 (0.1) %Male: 50.6% Age in years: Range: 12–19; Mean: 15.4	Serum 25(OH)D: radioimmunoassay SBP and DBP: mercury sphygmomanometer (not detailed)	For SBP: Age; Sex; Race/ethnicity; BMI For DBP: Age; Sex	● Lower SBP with increasing tertiles of vitamin D ● No association between vitamin D with DBP
Ghobadi, 2019- Shiraz, Iran [48]	240 children, without chronic disease, not on special diets, not taking drugs affecting metabolic status, recruited from elementary schools Mean BMI(SD) in kg/m^2^: 16.05 (2.74) %Males: 52.9% Age in years: Range: 6–9; Mean(SD): 7.8 (1.06)	Serum 25(OH)D: ELISA SBP and DBP: average of 2 readings, by mercury sphygmomanometer	Age; Gender; BMI; Physical activity	Inverse association between vitamin D with SBP and DBP
Ha, 2013- Suwon, South Korea [50]	310 children, free of disease not taking medications, recruited from elementary schools. Mean BMI(SD) in kg/m^2^; serum 25(OH)D Q1: 19.8 (3.7); Q2: 19.4 (3.7); Q3: 19.3 (3.3); Q4: 18.3 (3) %Male: 52% Age in years: Range:10–12; Mean (SD): Q1: 12.2 (1.2); Q2:12.2 (1.3); Q3: 12.3 (1.1); Q4:12.3 (1.1)	Serum 25(OH)D: chemiluminescence immunoassay SBP and DBP: average of 2 readings by an automated BP instrument	Age; Gender; Tanner stage; Body fatness; physical activity	● No differences in SBP and DBP across quartiles of vitamin D ● No association between vitamin D with SBP and DBP
Hannesdottir, 2017- Iceland [51]	159 elementary school children Mean BMI(SD) in kg/m^2^: NR %Male: 46.5% Age in years: Range:7	Serum 25(OH)D: radioimmunoassay Vitamin D status groups: Deficient: < 50 nmol/L; Sufficient: > 50 nmol/L SBP and DBP: average of 3 readings in a standard way using the left arm	None	● No association/correlation between vitamin D with SBP and DBP ● No differences in SBP and DBP across vitamin D status groups
Hassan, 2015- Egypt [52]	65 obese (BMI > 95th p for age and sex-specific growth curves) and 30 healthy (BMI between 15 and 85th p for age and sex-specific growth curves) children with no medical conditions, including type I and type II diabetes, not taking any medications that influence physical growth, recruited from primary public schools Mean BMI(SD) in kg/m^2^: 24.28 (5.95) %Male: 49.5% Age in years: Range: 8–11; Mean(SD): All: 9.99 (1.14); obese:10.1 (1.18) ; control: 10.27 (0.74)	Serum 25(OH) D: NR SBP and DBP: average of 2 readings by a mercury sphygmomanometer after a rest of 20 min	None	No association between vitamin D with SBP and DBP in the total sample, and in obese participants
Hirschler, 2012- Buenos Aires, Argentine [53]	116 boys without chronic disease, and not taking medications known to affect bone metabolism, recruited from amateur rugby club Mean BMI (SD) in kg/m^2^: 22.0 (5.3) %Male: 100% Age in years: Mean(SD): 11.3 (2.4)	Serum 25(OH)D: radioimmunoassay kit SBP and DBP: NR	Tanner stage	No differences in SBP and DBP across quartiles of vitamin D
Hirschler, 2013- Buenos Aires, Argentine [54]	290 Indian Koya children, free of chronic disease, recruited from schools Mean BMI in kg/m^2^: 16.88 (2.99) %Male: 44.5% Age in years: Range: 5–19; Mean(SD): 10.7(2.9)	Serum 25(OH)D: radioimmunoassay kit Vitamin D status groups: Deficient: < 50 nmol/L SBP and DBP: NR	Age (adjustment only for SBP across quartiles)	● Higher SBP and DBP with vitamin D deficiency ● Lower SBP with increasing quartiles of vitamin D
Hirschler, 2019- San Antonio de los Cobres and Chicoana, Argentine [55]	152 indigenous schoolchildren from San Antonio de los Cobres;175 from Chicoana not taking medications affecting BP, lipids, glucose level, with no significant difference in socioeconomic level, age, BMI and waist circumference, recruited from elementary schools Mean BMI(SD): San Antonio de los Cobres in kg/m^2^: 16.83 (2.69); Chicoana: 19.27 (4.41) %Male: 51.7% Age in years: Mean(SD) San Antonio de los Cobres: 9.37 (2.11); Chicoana: 9.02 (2.14)	Serum 25(OH)D: radioimmunoassay SBP and DBP: average of 2 readings, at a period of 1 to 2 min, by a mercury sphygmomanometer in a sitting position with the child’s right forearm horizontal on the table and cuffs sizes adjusted for differences in arm circumference and height HTN: average of the values of SBP and /or DBP ≥ 95th p based on age , sex and height p	Age; Gender; *z*-BMI; Milk intake	Inverse association between vitamin D with SBP and DBP
Hirschler, 2019- Argentine [56]	152 indigenous schoolchildren from San Antonio de los Cobres;175 from Chicoana not taking medications affecting BP, lipids, glucose level, with no significant difference in socioeconomic level, age, BMI and waist circumference, recruited from elementary schools Mean BMI(SD): San Antonio de los Cobres in kg/m^2^: 16.83 (2.69); Chicoana: 19.27 (4.41) %Male: 51.7% Age in years: Mean(SD) San Antonio de los Cobres: 9.37 (2.11); Chicoana: 9.02 (2.14)	Serum 25(OH)D: radioimmunoassay SBP and DBP: average of 2 readings at a period of 1 to 2 min by a mercury sphygmomanometer in a sitting position, with the child’s right forearm horizontal on the table and cuffs sizes adjusted for differences in arm circumference and height MAP: (DBP*2 + SBP) / 3	BMI; Age; Sex; Location; Triglycerides; Insulin; Glucose	No association between vitamin D with SBP, DBP and MAP
Izadi, 2020- Tehran, Iran [57]	514 students, without disease, or regular use of medication or supplement, recruited from local schools Mean BMI(SD) in kg/m^2^: 17.41 (0.19) % Male: 46.69% Age in years: Range: 7–12; Mean(SD): 9.16 (1.53)	Serum 25(OH)D: ELISA SBP and DBP: standard mercury pressure gauge with dimensions suitable for children and standard medical devices in a sitting position after a 5-min rest	Linear regression; Age; BMI; Gender; DBP; Triglycerides; HDL and Total Cholesterol	No association between vitamin D with SBP, in adjusted model
Jang, 2013- South Korea [58]	320 adolescents, not taking medications and not having high insulin levels, recruited from schools as part of the Korean Children Adolescent Study Not-overweight: 88.4%; Overweight: 11.5% % Male: 0% Age in years: Range: 12.4–14.5; Mean(SD): NR	Serum 25(OH)D: gamma counter with a radioimmunoassay Vitamin D status: Deficient: < 50 nmol/L SBP and DBP: average of 2 readings by a mercury sphygmomanometer in a sitting position at rest High BP: ≥ 130/85 mmHg	BMI *z*-score; Physical activity	● No correlation between vitamin D with SBP and DBP ● Higher SBP with vitamin D deficiency ● No differences in prevalence of HTN and DBP across vitamin D status groups
Kardas, 2013- Turkey [59]	114 children and adolescents obese (BMI ≥ 90th p for reference curves for Turkish children) and non-obese recruited from a children hospital Mean BMI(SD) in kg/m^2^: 24.5 (5.4); Mean BMI (obese): 28.5 (2.7); Mean BMI (non-obese); 19.6 (3.6) % Male: 50.9% Age in years: Mean(SD) obese: 13.5 (1.6); non-obese 13.4 (1.7)	Plasma 25(OH)D: HPLC SBP and DBP: average of 2 readings by a mercury sphygmomanometer, after a 20 min rest	None	Inverse correlation between vitamin D with SBP and DBP in the total sample; no longer significant when assessed among obese and non-obese participants
Kelishadi, 2014-Iran [61]	1095 nationally representative sample of Iranian students Mean BMI(SD) in kg/m^2^: 19.37 (4.58) % Male: 52% Age in years: Range: 10–18; Mean(SD): 14.74 (2.61)	Serum 25(OH)D: chemiluminescence immunoassay SBP and DBP: average of two readings by a standardized mercury sphygmomanometer in a sitting position on the right arm with an appropriate cuff size	Age; Gender; Anthropometric measures	Inverse association between vitamin D with SBP and DBP
Khadgawat, 2012- New Delhi, India [62]	62 obese Asian-Indian children and adolescents without any known systemic illness, endocrine or metabolic disorder or symptoms suggesting hypothalamic obesity, not taking any medications. Mean BMI(SD) in kg/m^2^: 29.3 (4.8) % Male: 56.5% Age in years: Range: 6–17; Mean(SD): 13 (3.1)	Serum 25(OH)D: radioimmunoassay Vitamin D status groups: Deficient:< 50 nmol/L; Severe; Deficiency: < 12.5 nmol/L; Moderate Deficiency: 12.5–25 nmol/L; Mild Deficiency :25–50 nmol/L SBP and DBP: average of 3 readings in a sitting position, after a 5 min rest ,by a mercury sphygmomanometer in the right upper arm with an appropriate size cuff	BMI; Age; Gender; Pubertal stage	No differences in SBP and DBP across vitamin D status groups
Kim, 2018- South Korea [64]	2314 adolescents selected from the KNHANES (2010–2014) nationwide survey who were fasting for more than 8 h, and had complete data for 25(OH)D and metabolic syndrome components Mean BMI(SD) in kg/m^2^: NR % Male: 53.80% Age in years: Range: 12–18; Mean(SD): NR	Serum 25(OH)D: NR Vitamin D status groups: Deficient: < 50 nmol/L; Sufficient: ≥ 50 nmol/L SBP and DBP: average of 3 readings on the right arm after a 5-min rest Elevated BP: SBP ≥ 130 mmHg; DBP ≥ 85 mmHg	Age; Gender; Household income; Residential area; Self-perceived health status; Self-perceived stress status; family history of chronic disease; Sleep habits, and physical activity habits	Similar odds of elevated BP across vitamin D status groups, in adjusted model
Kumar, 2009-USA [65]	9757 non-Hispanic white, non-Hispanic black, Hispanics/Mexicans and other race; children and adolescents from the NHANES study (2001–2004) Mean BMI(SD) in kg/m^2^: NR % Male: NR Age in years: Range: 1–21; Mean(SD): NR	25(OH)D: diasorin assay Vitamin D status groups: Deficient:< 37.5 nmol/L; Insufficient: 37.5–72.5 nmol/L SBP and DBP: average of 3 readings HTN: SBP or DBP >95th p for the median height of each participant's age and gender or > 140/90 mmHg in those ≥ 17 years of age	Age; Gender; Race/ethnicity; Poverty income ratio; Obesity; Milk intake; Television and computer use; vitamin D supplement use	● Higher odds of HTN with vitamin D deficiency ● Higher SBP and DBP with poorer vitamin D status
Lee, 2014-Seoul, South Korea [14]	1660 children from the KMOSES, no history of cardiovascular disease, diabetes, HTN or endocrine disorders, non-smoking, no alcohol consumption Mean BMI(SD) in kg/m^2^: Boys: 8.6 (3.4) Girls: 17.4 (3.0) % Male: 54.5% Age in years: Range: 9; Mean(SD): NR	Serum 25(OH)D: chemiluminescent immunoassay SBP and DBP: standard brachial cuff technique. HTN: > 90th p for sex, height and age	BMI	● No differences in prevalence of high BP, SBP and DBP across quartiles of vitamin D ● No difference in vitamin D level among normotensive and those with high BP
Lee, 2015- Seoul, South Korea [68]	2880 children and adolescents from the KNHANES (2008–2010) nationwide survey having data on 25(OH) D levels and blood sample for metabolic syndrome components Mean BMI(SD) in kg/m^2^: 20.46 (3.57) %Male: 53.4% Age in years: Range:10–18; Mean(SD): 13.74 (2.49)	25(OH)D: 125I-labelled radioimmunoassay kits SBP and DBP: average of 2 readings in the right arm, within 5 min interval, by a standard mercury sphygmomanometer at rest.	Age; Gender	Lower SBP and DBP with increasing quartiles of vitamin D
Lee, 2016- USA [69]	209 non-Hispanic white, non-Hispanic black, other race, overweight or obese patients recruited to participate in the OSCIR if having insulin resistance, depressed fasting glucose to fasting insulin ratio or referral from primary health clinics and free of chronic or acute infectious diseases, not taking glucose or lipid lowering medications. Mean BMI(SD): 35.9 (8.4) %Male: 45% Age in years: Range: 6–18; Mean(SD): 12.6 (2.9)	Plasma total 25(OH)D: in duplicates by Immunodiagnostic Systems enzyme immunoassay SBP and DBP: standard sphygmomanometer in a sitting position with an appropriate size cuff	None	No association between vitamin D with SBP and DBP
Malyavskaya, 2017- Russia [108]	319 children and adolescents, without acute and/or chronic diseases, recruited from secondary educational institutions Mean BMI(SD) in kg/m^2^: Q1: 22.6 (4.2); Q2: 20.8 (4.7); Q3: 20.2 (4.4); Q4: 19.6 (3.4) %Male: 51% Age in years: Range:10–15; Mean(SD): 13.3 (1.6)	Serum 25 (OH)D: ELISA SBP and DBP: NR	None	● No differences in SBP and DBP across quartiles of vitamin D, except for a higher DBP for Q 1 vs. 4 ● Inverse correlation between vitamin D with DBP only
Matter, 2016-KSA [72]	84 healthy adolescents attending an outpatient clinic, without acute or chronic disease or under treatment that could influence 25(OH)D level, and with 25(OH)D levels of < 50 or > 75 nmol/L Mean BMI(SD): NR % Male deficient group: 60%; control group:72% Age in years: Range: 12–16; Mean(SD) deficient group: 14.08 (2.01); control group: 13.93 (1.06)	Serum 25(OH)D: radioimmunoassay Vitamin D status: Deficient: < 50 nmol/L SBP and DBP: NR	None	No differences in SBP and DBP across vitamin D status groups
Mellati, 2015-Iran [73]	297 healthy schoolchildren Mean BMI(SD) in kg/m^2^: 17.81 (3.39) % Male: 45.10% Age in years: Range: 7–11 ; Mean(SD): 7.86 (1.32)	Serum 25(OH)D: ELISA using immunodiagnostic system SBP and DBP: average of 3 readings in 10 min intervals after at least a 15-min rest. For those with higher BP, the measurement was repeated on another day	None	● Lower SBP and DBP with increasing tertiles of vitamin D ● Inverse correlation between vitamin D with SBP and DBP
Milagres, 2017-Vicosa, Brazil [74]	378 children from all public and private schools from the Survey of Health Assessment of Schoolchildren, not taking medications interfering with metabolism of vitamin D, glucose, lipids, and no vitamin mineral supplementation. Mean BMI Z-score: 0·41 (1.4) % Male: 47.8% Age in years: Range: 8–9; Mean(SD): NR	Serum 25(OH)D: architect 25-OH vitamin D assay Vitamin D status group: Deficient: < 50 nmol/L; Insufficient: ≥ 50–< 75 nmol/L; Sufficient: ≥ 75 nmol/L SBP and DBP: average of 3 readings by an automatic inflation BP monitor in a sitting position with at least a 5-min rest Elevated BP: SBP or DBP ≥ 90th p for age, gender and height according to the VI Brazilian guidelines of HTN by the Brazilian Society of Cardiology	Age; Gender; Season Ethnicity; PTH; Per capita income; Maternal schooling; vitamin D intake; Sedentary behavior; Percentage of body fat (or other measures of adiposity)	No difference in prevalence of elevated BP and HTN across vitamin D status groups
Moore, 2017-USA [75]	2908 children and adolescents from the NHANES study (2007–2010), not pregnant, and completed 24-h recall Mean BMI(SD) in kg/m^2^: NR % Male: 51.4% Age in years: Range: 8–18; Mean(SD): NR	Serum 25(OH)D: LC/MS Vitamin D status group: Deficient: < 50 nmol/L; Insufficient: 50–72.5 nmol/L; Sufficient :> 72.5 nmol/L SBP and DBP: average of 3 readings by the auscultatory method in a sitting position for 5 min Normal BP: SBP or DBP < 90th p for age, sex and height; Pre-HTN: SBP: ≥ 90th to < 95th p for age, sex and height; HTN: ≥ 95th p for age, sex and height	Race/ethnicity; Sex; Age; Economic status; BMI *z*-score	● No association between vitamin D with SBP and DBP, in adjusted model ● Higher prevalence of HTN with poorer vitamin D status
Muhairi, 2013- Al Ain, UAE [76]	315 healthy adolescents (BMI: 5–75th p for age and sex), not using regular medications or have chronic medical conditions that might affect growth, body composition, dietary intake or physical activity and non-smokers, recruited from schools obese:16.5%; Overweight :15.9; Lean: 67.5% % Male: 48% Age in years: Range: 12–18; Mean(SD): NR	Serum 25(OH)D: radioimmunoassay SBP and DBP: average of 2 readings by a standard mercury sphygmomanometer after a 5-min rest in a sitting position with appropriate cuff size	None	No difference in vitamin D level among normotensive and those with high BP
Nam, 2012- South Korea [77]	713 adolescents from the KNHANES (2007–2009), nationwide survey not having congenital heart disease or previous diagnosis of epilepsy Mean BMI (SD) in kg/m^2^: 21.12 (0.16) % Male: 53.01 Age in years: Range: 12–19; Mean(SD): NR	Serum 25(OH)D: radioimmunoassay SBP and DBP: average of 2 measurements in 5-min intervals, by standard mercury sphygmomanometer on the right arm	None	● No difference in SBP across tertiles of vitamin D ● Lower DBP with increasing tertiles of vitamin D
Nam, 2014- South Korea [78]	1504 adolescents from the KNHANES (2008–2009), nationwide survey, not having congenital heart disease or epilepsy Mean BMI(SE): insufficient group: 21.2 (0.1); sufficient group: 20.4 (0.2) % Males: 52.65% Age in years: Range: 12–18; Mean(SD): NR	Serum 25(OH)D: radioimmunoassay Vitamin D status groups: Insufficient: ≤ 50 nmol/L; Sufficient: > 50 nmol/L SBP and DBP: average of 2 measurements in 5-min intervals, by standard mercury sphygmomanometer on the right arm High BP: SBP or DBP ≥ 90th p for age and sex, use of BP-lowering medication or a previous diagnosis of HTN	For the OR between high BP and 25(OH)D: Age; Gender; BMI; Regular physical exercise; Alcohol drinking; Use of multivitamin or mineral supplements	● Inverse correlation between vitamin D with SBP and DBP ● No difference in SBP across vitamin D status groups; higher DBP with vitamin D insufficiency ● Similar odds of high BP across vitamin D status groups, in adjusted model
Nsiah-Kumi, 2012- Nebraska, USA [80]	198 native American healthy youth (without active infection, or illness that could affect weight), recruited from schools, local grocery store, tribal exercise facility, health facilities, community events Mean BMI p: 78 (1.7) % Male: 47% Age in years: Range: 5–19; Mean(SEM): 10.8 (0.3)	25(OH)D: radioimmunoassay vitamin D status groups: Deficient: < 40 nmol/L; Insufficient: < 75 nmol/L SBP and DBP: after a 5-min rest, using an appropriately sized cuff	BMI p for age and sex	Inverse association between vitamin D with SBP and DBP
Nwosu, 2013-Central New England, USA [81]	45 prepubertal healthy children recruited using paper flyers from primary care physician offices Normal weight: 44.4%; Overweight: 55.6% % Male: 58% Age in years: Range: 3–12; Mean(SD): 8.3 (2.5); Males: 9.0 (2.4); Females: 7.28 (2.4)	Serum 25(OH)D: chemiluminescent immunoassay Vitamin D status groups: Deficient:< 50 nmol/L; Sufficient: > 50 nmol/L SBP and DBP: NR	None	No differences in SBP and DBP across vitamin D status groups
Oliveira, 2014-Juiz de Fora, Brazil [83]	160 healthy adolescents not using medications or supplements, recruited from schools Normal weight: 48.1%; Overweight: 51.9% (matched for age, gender and type of school) % Male: 55.6% Age in years: Range: 15–17; Mean(SD): 16 (0.9)	Serum 25(OH)D: radioimmunoassay SBP and DBP: average of the second and third measurements, at 5-min intervals, with the right arm at the same level as the heart, by an equipment validated against mercury sphygmomanometers according to the international validation protocol, using an appropriate cuff size, in a sitting position Elevated BP: based on parameters of the Brazilian HTN Society taking into account gender, age and height	None	Lower vitamin D level among hypertensive participants
Olson, 2012- North Texas, USA [84]	411 obese (BMI ≥ 95th p for age) children without disease, not using medications or vitamin D supplement > 400 IU/day, recruited from an obesity center at a children’s medical center Mean BMI(SD) in kg/m^2^: NR % Male: 43% Age in years: Range: 6–16; Mean(SD): 11.7(2.6)	25(OH)D: chemiluminescent immunoassay SBP and DBP: average of up to 3 measures, by dinamap procare monitor, at rest	BMI *z*-scores; Age	No correlation between vitamin D with SBP and DBP
Pacifico, 2011-Rome, Italy [85]	452 healthy children recruited from outpatient clinics %Male Tertile 1: 49.4%; Tertile 2: 45%; Tertile 3: 45.6% Overweight/obese: 67.25% Age in years: Median(IQR): Tertile 1: 11.5 (4.2); Tertile 2: 11.2 (4.3); Tertile 3: 11.0 (4.0)	Serum 25(OH)D3: electrochemiluminescence immunoassay SBP and DBP: average of 2 measures, at the right arm in the supine position using an automated oscillatory system, after a 10-min rest Elevated BP: SBP or DBP ≥ 90th p for age, gender and height	For correlation: age; gender; Tanner stage For regression: Model 1: age; gender; Tanner stage Model 2: age; gender; Tanner stage; waist circumference Model 3: age; gender; Tanner stage; Standard deviation score-BMI	● Lower SBP and DBP with increasing tertiles of vitamin D ● Negative correlation between vitamin D with SBP only ● Lower odds of elevated BP with increasing tertiles of vitamin D, in adjusted model
Parikh, 2012- Augusta area, USA [86]	701 healthy adolescents, not taking medications, recruited from high schools Mean BMI(SD) in kg/m^2^: 23.0 (4.7) Age in years: Range: 14–18; Mean(SD): 16.2 (1.2)	Plasma 25(OH)D: liquid chromatography tandem mass spectroscopy SBP and DBP: NR	Age; Gender; Ethnicity; Sexual maturation; Season; Physical activity; Percent body fat	● Inverse correlation between vitamin D with SBP and DBP ● Lower SBP with increasing tertiles of vitamin D; No difference in DBP
Petersen, 2015-Denmark [87]	782 healthy children, not taking medications, recruited from schools Girls: Obese: 2%; Overweight: 13%; Normal weight: 74%; Underweight: 11% Boys: Obese: 2%; Overweight: 11%; Normal weight: 78%; Underweight: 9% Age in years: Range: 8–11; Mean(SD): Boys: 10.1 (0.6); Girls: 9.9 (0.6)	Serum 25(OH)D (including both D2 and D3): automated chemiluminescent immunoassay SBP and DBP: average of 3 readings, after 10-min rest, by an automated device, using two different cuff sizes	Sex; Age; Height; Ethnicity; Whole-blood EPA+ DHA; Entered puberty (yes/no); Parental education	● No association between vitamin D with SBP ● Inverse association with DBP, in adjusted model
Pirgon, 2013-Turkey [88]	87 obese adolescents, recruited from a pediatric endocrinology unit: 45 patients with NAFLD and 42 patients without NAFLD, not taking medications, and free of other diseases Mean BMI(SD) in kg/m^2^: 2.1 (0.3) % Male: 48.2% Age in years: Range: 11–15; Total: Mean(SD): 12.7 (1.3); NAFLD group:12.8 (0.8); Non-NAFLD group:12.6 (1.7)	Serum 25(OH)D: automated chemiluminescence immunoassay SBP and DBP: mercury-gravity manometer and a cuff appropriate for body size, in a sitting position, after rest for at least 5 min	None	No correlation between vitamin D with SBP and DBP in participants with and without NAFLD
Prodam, 2016- Novara area, Italy [89]	575 healthy, overweight or obese, sedentary children and adolescents (according to International Obesity Task Force criteria), not using medications, recruited from a pediatric endocrinology clinic Mean BMI (SD) in kg/m^2^: 26.7 (4.5) Severely obese: 27.6%; Obese: 44.2%; Overweight: 28.2% % Male: 50.26% Age in years: Range: 6–18; Mean(SD): 10.7(2.8)	Serum 25(OH)D: direct competitive chemiluminescent immunoassay SBP and DBP: average of 3 measurements on the left arm, after a 15-min rest in the supine position and prior to other physical evaluations, using a standard mercury sphygmomanometer	None	Inverse correlation between vitamin D and SBP only
Rafraf, 2014- Boukan, Iran [90]	216 healthy adolescents, not using medication or supplements, recruited from high schools Mean BMI(SD) in kg/m^2^: 21.1 (3.5) % Male: 0% Age in years: Range: 14–17; Mean(SD): 15.9 (1.0)	Serum 25(OH)D: ELISA SBP and DBP: average of 2 measurements, at a 1–2 min interval, in the morning by a mercury sphygmomanometer with an adult cuff on the upper right arm, with the arm horizontally on a table, in the sitting position, after a 5-min rest	BMI; Energy; Physical activity level	No association between vitamin D with SBP and DBP
Reis, 2009- USA [91]	3528 adolescents nationally representative sample of white, black, Mexican American and other race (NHANES 2001–2004) not diabetic, not pregnant Mean BMI(SD) in kg/m^2^: NR % Male: 51.5% Age in years: Range: 12–19; Mean(95%CI): 15.4 (15.3–15.6)	Serum 25(OH)D: radioimmunoassay SBP and DBP: average of up to 4 measures by a mercury-gravity sphygmomanometer using appropriate arm cuff size, in seated position, after a 5-min rest High BP: SBP or DBP ≥ 90th p for age, sex, and height, or use of BP medications	Age; Gender; Ethnicity; BMI Poverty-to-income ratio; Physical activity No adjustment for vitamin D as outcome	● Lower prevalence of high BP and SBP with increasing quartiles of vitamin D; No difference in DBP ● Lower vitamin D level among participants with high BP
Simpson, 2020-Connecticut, USA [92]	203 healthy, urban-dwelling children, not using medications or vitamin D supplements > 400 IU/day, recruited to participate in a vitamin D supplementation study from local medical offices and community site Mean BMI(SD) in kg/m^2^: NR % Male: 50.24% Age in years: Range: 6 months–10 years; Mean(SD): 5.6 (2.3)	Total serum 25-OHD and 1,25(OH)2D: radioimmunoassay Calculated free 25(OH)D: calculated using serum vitamin D binding protein and albumin concentrations, and their reported dissociation constants Genotype-specific free 25(OH)D: calculated using 25(OH)D/DBP dissociation constants specific for each individual’s haplotype Direct measured free 25(OH)D: ELISA SBP and DBP: NR	None	Inverse correlation between vitamin D with SBP only
Skrzypczyk, 2018- Poland [93]	49 children and adolescents with primary HTN, not using vitamin D supplements during last 12 months Obese: 30.6%; Overweight: 28.6% % Male: 69.4% Age in years: Range: 5.58–18; Mean(SD): 14.29 (3.17)	25(OH)D: chemiluminescence Peripheral BP: using oscillometric device 24-h BP: using a SUNTECH OSCAR 2 device and interpreted according to the American Heart Association guidelines. Monitors were programmed to measure BP every 15 min from 6 AM to 10 PM and every 30 min from 10 PM to 6 AM. SBP, DBP and MAP: measured during 24 h	None	No correlation between vitamin D with SBP and DBP
Teixeira, 2018- Rio de Janeiro, Brazil [96]	60 severely obese adolescents (BMI > 99.9th p for age), without chronic disease, not taking medications or vitamin D supplementation, recruited from an obesity clinic Mean BMI(SD) in kg/m^2^: 46.21 (7.01) % Male: 36.7% Age in years: Range: 10–20; Mean(SD): 17.32 (1.35)	Serum 25(OH)D: HPLC Vitamin D status group: Deficient: ≤50 nmol/L; Insufficient: > 50– < 75 nmol/L; Adequate: 75–247 nmol/L SBP and DBP: average of 2 measures taken 1 min apart, by oscillometric technique semi-automatic digital arm device, after a 5-min rest HTN: according to the VI Brazilian Guidelines for HTN in adolescents	None	No difference in prevalence of HTN across vitamin D status groups
Tomaino, 2015- Lima and Tumbes, Peru [97]	1074 adolescents selected from a community census Mean BMI in kg/m^2^: 21.2 Overweight (based on standard recommendations for age- and sex-specific body mass index cutoffs for international, adolescent populations): 22% % Male:48% Age in years: Range: 13–15; Mean(SD): 14.9 (0.8)	25(OH)D: in duplicate, using the LIASON 25-OH vitamin D total assay Vitamin D status group: Deficient: < 50 nmol/L; Non-deficient: ≥ 50 nmol/L SBP and DBP: median of 3 measurements, after 5-min rest, using the right arm and in the seated position MAP: 1/3 SBP + 2/3 DBP	Overweight status; Age; Sex; Height; Seasonality; Personal smoking status; Second-hand smoke exposure; Monthly household income; Study site	Higher MAP, SBP, DBP with vitamin D deficiency
Valle, 2019- Rio de Janeiro, Brazil [98]	97 overweight (BMI ≥ 85th p) and obese adolescents (BMI ≥ 95th p), cared for in the NESA, free of chronic disease, not taking medications or supplements Mean BMI(SD) in kg/m^2^: 32.2 (6.3) %Male: 44% Age in years: Range: 12–19; Mean(SD): 14.7 (1.8)	Serum 25(OH)D: HPLC Vitamin D status group: Deficient: < 50 nmol/L SBP and DBP: by an automatic inflation BP monitor (not detailed) High BP: NR	None	● Inverse association between vitamin D with DBP only ● Higher prevalence of elevated BP with vitamin D deficiency ● Higher SBP with vitamin D deficiency
Williams, 2011-USA [100]	Nationally representative sample of healthy adolescents (NHANES 2001–2006) [N for 25(OH)D: 6013; SBP: 4807; DBP: 4777] % Male: Quintile 1: 40.7%; Quintile 2: 52.9%; Quintile 3: 53.7%; Quintile 4: 54.6%; Quintile 5: 54.6% Age in years: Range: 12–19; Mean(95%CI): Quintile 1: 15.7 (15.5, 15.9); Quintile 2: 15.1 (14.9, 15.3), Quintile 3: 14.9 (14.7, 15.2); Quintile 4: 15.2 (15.0, 15.5); Quintile 5: 15.6 (15.4, 15.8)	Serum 25(OH)D: radioimmunoassay SBP and DBP: average of up to 4 measurements, at rest	Age; Gender; Ethnicity; Poverty income ratio; Waist circumference; Sampling probability (via weights); Cluster effects	Inverse association between vitamin D and SBP only
Wojcik, 2017- Krakow, Poland [104]	30 obese adolescents recruited from a children’s university hospital Mean BMI(SD)in kg/m^2^: 32.5 (4.85) % Male: 46.66% Age in years: Range: NR; Mean(CI): 13.23 (12.64–13.8)	Serum 25(OH)D: HPLC Vitamin D status group: Deficient: < 50 nmol/L SBP and DBP: average of 3 measurements, every 3 min, using a pneumatic sphygmomanometer Arterial HTN: mean SBP and/or DBP >95th p for age, height and gender	None	Higher prevalence of arterial HTN and DBP with vitamin D deficiency; no difference in SBP
Xiao, 2020- China [105]	6091 nationally representative sample of children and adolescents, without any condition, or use of drug affecting cardiovascular health, recruited from schools Mean BMI(SD) in kg/m^2^_:_ NR % Male:50.2% Age in years: Range: 6–18; Mean(SD): 11.9 (3.7)	Plasma 25(OH)D: chemiluminescent immunoassay Vitamin D status group: Adequacy: < 50 nmol/L; Inadequacy: > 50 nmol/L SBP and DBP: average of last 2 reading out of 3, with 1–2 min intervals, after resting for at least 15 min, in a sitting position from the right arm using a suitable cuff size based on the arm circumference HTN: average SBP and/or DBP ≥ 95th sex, age and height-specific p for Chinese children and adolescents, or taking antihypertensive drugs	Age; Gender; Season of blood collection; Geographical location; Smoking; Drinking; Physical activity; Dietary vitamin D intake; BMI; Fat mass percentage; Muscle mass index	Higher odds of HTN with vitamin D inadequacy, in the total sample and in girls only
Yousefichaijan, 2019- Arak, Iran [106]	65 healthy children, without disease, not taking hypertensive drugs, with vitamin D deficiency, recruited from a hospital Mean BMI(SD) in kg/m^2:^ 16 (2.84) % Male: 49.2% Age in years: Range < 11; Mean(SD): 13.9 (3.2)	Serum 25(OH)D: NR Vitamin D status group: Deficient: < 50 nmol/L SBP and DBP: digital monitor citizen Hypertensive status: Normal: BP < 90th p for age, gender and height; Pre-HTN: BP: 90–95th p; Stage 1 HTN: BP: 95–99th p + 5 mmHg; Stage II HTN: BP: >99th pe + 5 mmHg	None	No difference in vitamin D level across BP status groups
Zhou, 2011- USA [107]	140 healthy obese (BMI > 95th p for age and gender) children, recruited from a pediatric endocrine clinic Mean BMI(SD) in kg/m^2^: 34.5 (7.4) % Male: 40.7% Age in years: Range: 6–21; Mean(SD): 13.9 (3.2)	Serum 25(OH)D: chemiluminescent assay SBP and DBP: NR	Age	● Negative correlation between vitamin D with SBP only ● Higher SBP with vitamin D deficiency
**Retrospective**				
Aypak, 2014- Ankara- Turkey [39]	168 medical records of Turkish children, from outpatient pediatric clinics Obese: 26.2%; Overweight 20.2%; Lean: 53.6% % Male: 51.8% Age in years: Range: 4–16; Median: 11	25(OH)D: imunochemiluminescent assay SBP and DBP: NR	None	No correlation between vitamin D with SBP and DBP
Gul, 2017- Tokat- Turkey [49]	310 obese children (> 95th p for sex-specific growth curves and cut-off levels for Turkish children), followed up at an obesity clinic Mean BMI(SD) in kg/m^2^: 29.22 (4.71) % Male: 42.6% Age in years: Range: 6–17; Mean(SD): 12.10 (2.82)	Serum 25(OH)D: chemiluminescence immunoassay Vitamin D status groups: Deficient: < 37.5 nmol/L; Insufficient: 37.5–72.5 nmol/L; Sufficient: ≥ 75 nmol/L SBP and DBP: NR HTN: BP ≥ 95th p for age, sex and height	None	● No differences in HTN frequency, SBP and DBP according to vitamin D status groups ● No correlation between vitamin D with SBP and DBP ● Lower vitamin D level among hypertensive participants
Kao, 2015- Australia [16]	229 obese children and adolescents (BMI ≥ 95th p for age and sex-specific growth curves) without pre-existing disorders of vitamin D synthesis or action, attending obesity outpatient clinics Mean BMI(SD) in kg/m^2^: 34.8 (94) % Male: 50.7% Age in years: Range: 2–18; Mean(SD): 12.1 (3)	Serum 25(OH)D: electrochemiluminescent immunoassay or direct chemiluminescence competitive immunoassay SBP and DBP: manual sphygmomanometer in a seated position with appropriate cuff size	BMI; Age; Gender; Season	● Lower SBP and DBP with increasing quintiles of vitamin D ● Higher odds of elevated BP with lower quintiles of vitamin D
Kumaratne, 2017- California, USA [66]	234 healthy Hispanic adolescents from pediatric clinics not taking vitamin D supplementation and having no chronic illness by chart review BMI (< 85th p): 44.4%; Overweight(≥ 95th p for age and gender); Obese (BMI > 85th p for age and gender): 55.6% % Male: 53% Age in years: Range: 13–19; Mean(SD): NR	Serum 25(OH)D: NR Vitamin D status groups: Deficient: < 50 nmol/L; Adequate: ≥ 50 nmol/L SBP and DBP: NR	None	No differences in SBP and DBP across vitamin D status groups, among all weight categories
MacDonald, 2017- Alberta, Canada [71]	217 overweight (BMI 85–97 p for age and sex)and obese (> 97th p for age and sex) children attending a pediatric weight management clinic, data collected from charts Mean BMI(SD) in kg/m^2^: Deficient group: 33.0 (8.1); Sufficient group: 30.1 (6.6) % Male: 50% Age in years: Range: 12–18; Mean(SD): 12.0 (2.9)	Serum 25(OH)D: according to standard methodologies Vitamin D status groups: Deficient: < 50 nmol/L; Sufficient: > 50 nmol/L SBP and DBP: by an automatic BP machine with appropriate cuff size	None	No differences in SBP and DBP across vitamin D status groups
Smotkin-Tangorra, 2007- New York, USA [95]	217 obese (>95th p for age and sex) children and adolescents, recruited from a pediatric endocrine clinic at an infants and children's hospital Mean BMI(SD) in kg/m^2^: 32.2 (6.4) % Male: 45.6% Age in years: Range: 7–18; Mean(SD): 12.9 (5.5)	25(OH)D: NR Vitamin D status groups: Insufficient: < 50 nmol/L SBP: 1 measure in seated position	None	Higher SBP with vitamin D insufficiency
Williams, 2014- Pennsylvania, USA [103]	150 obese children and teenagers (≥ 95 p) attending a pediatric weight loss program Mean BMI(SD) in kg/m^2^: NR % Male: 35% Age in years: Range: 5–19; Mean(SD): Deficient group: 14.5 (3.1); Insufficient group: 13.6 (3.4)	Serum 25(OH)D: NR Vitamin D status groups: Deficient: < 50 nmol/L; Insufficient: < 75 nmol/L SBP and DBP: NR High BP: > 95th p for age and sex	Age; Sex; Race; Location; Season; Insulin level; Hyperlipidemia; Total comorbidities	Higher SBP with poorer vitamin D status; no difference in prevalence of high BP and DBP
**Case-control**				
Liang, 2018- China [70]	164 children from an established cohort recruited from elementary schools divided into: Hypertensive group: children diagnosed with HTN, not under treatment for vitamin deficiency and not taking antihypertensive drugs or other medications, and free of other diseases Non-hypertensive group: children without HTN or any other disease that affect vitamin absorption and metabolism Mean BMI(SD) in kg/m^2^:19.45 (4.59); hypertensive group: 22.51 (4.39); non-hypertensive group: 16.36 (1.94) % Male: 50.6% Age in years: Range: 6–12; Mean(SD): 9.81 (1.62)	Serum 25(OH)D: HPLC SBP and DBP: average of 3 readings in the sit-down position by electronic sphygmomanometer using an appropriately sized BP cuff placed on the right arm	BMI	Lower vitamin D level among participants with HTN; NS after adjustment
**Interventional (baseline assessment)**				
Al Daghri, 2016- Riyadh, Saudi Arabia [109]	77 children and adolescents, overweight/obese, with fasting blood glucose 5.6–6.9 nmol/L, without acute or chronic medical conditions, not taking vitamin D supplements, recruited from public schools and health centers Mean BMI(SD) in kg/m^2^: 32.31 (5) % Male: 33.5% Age in years: Range: 12–17; Mean(SD): NR	Serum 25(OH)D: COBAS e-411 automated analyzer SBP and DBP: average of 2 readings, at rest	None	Inverse correlation between vitamin D with DBP only
Khayyatzadeh, 2018- Iran [63]	988 healthy adolescents not taking medications or supplements Mean BMI(SD) in kg/m^2^: 21.07 (4.2) % Male: 0% Age in years: Mean(SD): Deficient group: 14.5 (1.53); Insufficient group: 14.7 (1.51); Sufficient group: 15.2 (1.53)	Serum 25(OH)D: electrochemiluminescence Vitamin D status groups: Deficient: < 50 nmol/L; Insufficient :50–74.9 nmol/L; and Sufficient: > 75 nmol/L SBP and DBP: standard procedure	None	No differences in SBP and DBP across vitamin D status groups
Ohlund, 2020- Umea and Malmo, Sweden [82]	206 healthy children recruited for a vitamin D supplementation study Mean BMI(SD) in kg/m^2^: NR % Male: 46.1% Age in years: Range: 5–7; Mean(SD): NR	Serum 25(OH)D2 and 25(OH)D3: MS on an API 4000 LC/MS/MS system (AB Sciex) SBP and DBP: using an automated oscillometric sphygmomanometer in Umea, and an automatic BP monitor in Malmo	Gender; Skin color; Study site; Mothers’ education	Inverse association between vitamin D with SBP and DBP, in adjusted model
Smith, 2018- United Kingdom [94]	110 healthy adolescents, not taking vitamin D supplement or planning a winter sun vacation Obese (≥ 95th p for age): 7%; Overweight (≥ 85– < 95th p for age): 12%; Normal (≥ 2nd– < 85th p for age): 81% % Male: 43% Age in years: Range: 14–18; Mean(SD): 15.9 (1.4)	Serum 25(OH)D: liquid chromatography-tandem mass spectrometry SBP and DBP: average of 3 measurements, 1-min apart, using an automatic BP monitor, in upright position with the arm supported	Sex; Age; BMI *z*-score; Tanner stage; Physical activity	No association between vitamin D with SBP and DBP
**Cross-sectional baseline assessments from prospective cohort studies**				
Kwon, 2015- South Korea [67]	205 prepubertal children from Ewha Birth and Growth Cohort Study Mean BMI(SD) in kg/m^2^: NR % Male: 53.2% Age in years: Range: 7–9; Mean(SD):7.89 (0.85)	Serum 25(OH) D: radioimmunoassay SBP and DBP: average of 2 readings, 5 min apart, by an automatic device with the correct cuff size and the arm properly supported	Age; Sex; BMI *z*-score; Birth order; Fruit/fruit juice intake; Maternal educational level	No association between vitamin D with SBP and DBP, in adjusted model
Williams, 2012- Avon, Southwest England [101]	4274 children from the Avon Longitudinal Study of Parents and Children at 9 years of follow-up Mean BMI(SD) in kg/m^2^: 17.6 (2.7) % Male: NR Age in years: Mean(SD): 9.86 (0.32)	25(OH)D: HPLC Season-adjusted 25(OH)D3 Total 25(OH)D: sum of 25(OH)D2 and unadjusted 25(OH)D3 SBP and DBP: mean of 2 measurements, using a Vital Signs monitor, at rest, with the arm supported at chest level	Age; Gender; Ethnicity; Socioeconomic position; Waist circumference; PTH; Circulating calcium and phosphate level	No association between vitamin D with SBP and DBP, in adjusted model
**Cross-sectional baseline assessment from a prospective cohort study and cross-sectional**				
Nandi-Munshi, 2017- USA [79] NHANES (2001–2006): Cross-sectional SNAS: Prospective cohort (baseline assessment)	NHANES: 8789 children and adolescents Obese: 19.79%; Overweight: 16.48%; Lean: 63.73% % Male: 49.9% Age in years: Range: 6–19 SNAS: 938 youth with type 1 diabetes Obese: 13.02%; Overweight: 21.04%; Lean: 65.94% % Male: 52.2% Age in years: up to 19	NHANES: Serum 25(OH)D: radioimmunoassay SBP and DBP: average of up to 3 measures, at rest, using a mercury sphygmomanometer BP: normal: SBP and DBP <90th p; pre-HTN: SBP or DBP ≥ 90th–< 95th p; HTN: SBP or DBP ≥ 95th p SNAS: Serum 25(OH)D: chemiluminescence immunoassay based on a linkage between specific vitamin D antibody-coated magnetic particles and an isoluminol derivative SBP and DBP: average of 3 measures, using a mercury sphygmomanometer HTN: BP > 90 p for age, sex, and height, or use of antihypertensive drugs	None	No difference in vitamin D level among normotensive and those with HTN

*BMI* body mass index, *SD* standard deviation, *25(OH)D* 25-hydroxyvitamin D, *ELISA* enzyme-linked immunosorbent assay, *SBP* systolic blood pressure, *DBP* diastolic blood pressure, *NR* not reported, *USA* United States of America, *p* percentile, *BP* blood pressure, *EPITeen* EPIdemiological health Investigation of Teenagers in Port, *HELENA* Healthy Lifestyle in Europe by Nutrition in Adolescence, *HTN* hypertension, *NESA* Nucleo de Estudos da Saude do Adolescente, *HPLC* high-performance liquid chromatography, *NHANES* National Health and Nutrition Examination Survey, *Q* quartile, *MAP* mean arterial pressure, *HDL* high-density lipoprotein cholesterol, *KNHANES* Korean National Health and Nutrition Examination Survey, *KMOSES* Korean Metabolic Disorders and Obesity Study in Elementary School Children, *OSCIR* Oxidative Stress in Childhood Insulin Resistance, *PTH* parathyroid hormone, *LC/MS* liquid chromatography/mass spectrometry, *SE* standard error, *SEM* standard error to the mean, *IU* international unit, *IQR* interquartile range, *EPA* eicosapentaenoic acid, *DHA* docosahexaenoic acid, *NAFLD* non-alcoholic fatty liver disease, *1,25(OH)2D* 1,25-dihydroxyvitamin D, *CI* confidence interval

#### Results of the studies

The findings of non-prospective cohort studies are also available in Table 2 (see Additional file 3 for the detailed numerical results of the studies). Among the 36 studies [27–34, 38–41, 43, 44, 47, 49, 51, 52, 59, 63, 66, 69, 71–73, 77, 81, 88, 89, 92, 93, 95, 96, 98, 104, 108, 109] that did not adjust for potential confounders, ten [28, 29, 31, 47, 73, 89, 92, 95, 98, 109] found a significant inverse association between vitamin D and SBP (of which three [29, 31, 109] in boys only), while the majority (*n* = 25) [30, 32–34, 38–41, 43, 44, 49, 51, 52, 59, 63, 66, 69, 71, 72, 77, 78, 88, 93, 104, 108] did not report such findings. Regarding the relationship between vitamin D and DBP, twelve [28–32, 47, 73, 77, 98, 104, 108, 109] found a significant inverse association (of which two [31, 109] in boys only), whereas most of the studies (*n* = 21) [33, 34, 38–41, 44, 49, 51, 52, 59, 63, 66, 69, 71, 72, 81, 88, 89, 92, 93] did not. Regarding hypertension, three studies [47, 98, 104] found higher prevalence with poorer vitamin D status. In contrast, two studies [49, 96] did not report any relationship between vitamin D and hypertension status.

Regarding the four studies [46, 54, 68, 107] that adjusted for age and/or gender, all of them [46, 54, 68, 107] found a significant inverse association between vitamin D and SBP. Also, three [46, 54, 68] reported a negative relationship with DBP; yet, only one study [107] did not find any association between vitamin D and DBP. One study [46] reported on higher prevalence of hypertension with lower vitamin D levels. The only study [58] that adjusted for BMI and physical activity found a higher SBP with vitamin D deficiency, but did not find any association between vitamin D and DBP, as well as the prevalence of hypertension.

Out of the six studies [14, 35, 37, 61, 80, 84] which adjusted for age, gender, and/or anthropometric measurements including BMI, three [35, 61, 80] found a significant inverse association between vitamin D and SBP, whereas another three [14, 37, 84] did not find such a relationship. Two studies [61, 80] reported a significant negative association between vitamin D and DBP, whereas four [14, 35, 37, 84] did not. The only study [14] that assessed high BP did not report any significant association with vitamin D.

Regarding the three studies [53, 62, 85] that adjusted for age, gender, anthropometric measurements, and/or sexual maturation level, one [85] found a significant inverse association with SBP, while two [53, 62] did not find such an association. Further, one [85] found a significant inverse association with DBP, whereas two [53, 62] did not find any association. The only study [85] that assessed high BP status, reported an inverse relationship with vitamin D.

Regarding the 27 studies [15, 16, 36, 42, 45, 48, 50, 55–57, 64, 65, 67, 74, 75, 78, 82, 86, 87, 90, 91, 94, 97, 100, 101, 103, 105] which adjusted for multiple confounding factors, eleven studies [15, 16, 48, 55, 65, 82, 86, 91, 97, 100, 103] reported a significant inverse relationship between vitamin D and SBP, whereas twelve [36, 42, 45, 50, 56, 57, 67, 75, 87, 90, 94, 101] found no such relationship. Eight studies [16, 48, 55, 65, 82, 86, 87, 97] found a significant inverse relationship between vitamin D and DBP, yet, the majority (*n* = 14) [15, 36, 42, 45, 50, 56, 67, 75, 90, 91, 94, 100, 101, 103] found no such association. Finally, six [16, 65, 75, 78, 91, 105] found an inverse association between vitamin D and elevated BP status, whereas three [64, 74, 103] did not.

Only eleven studies [14, 42, 43, 49, 70, 76, 79, 83, 91, 99, 106] investigated vitamin D levels across groups of BP status. Among them, four studies [49, 70, 83, 91] found a lower vitamin D level in participants with elevated BP compared with their counterparts; whereas seven [14, 42, 43, 76, 79, 99, 106] did not find a difference in vitamin D level among normotensive participants and those with high BP.

#### Assessment of risk of bias

**Fig. 3:**
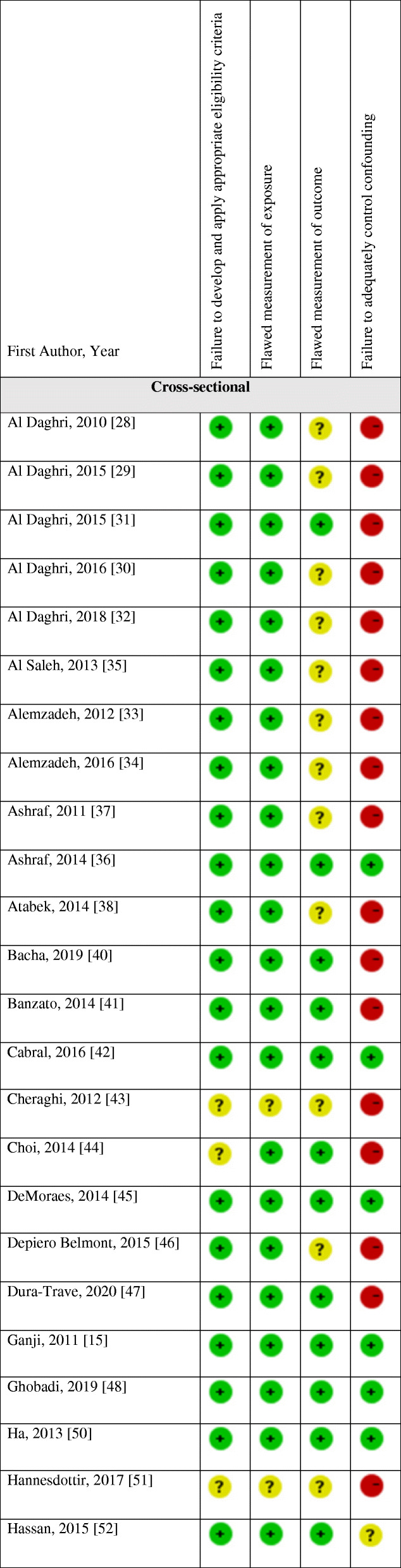

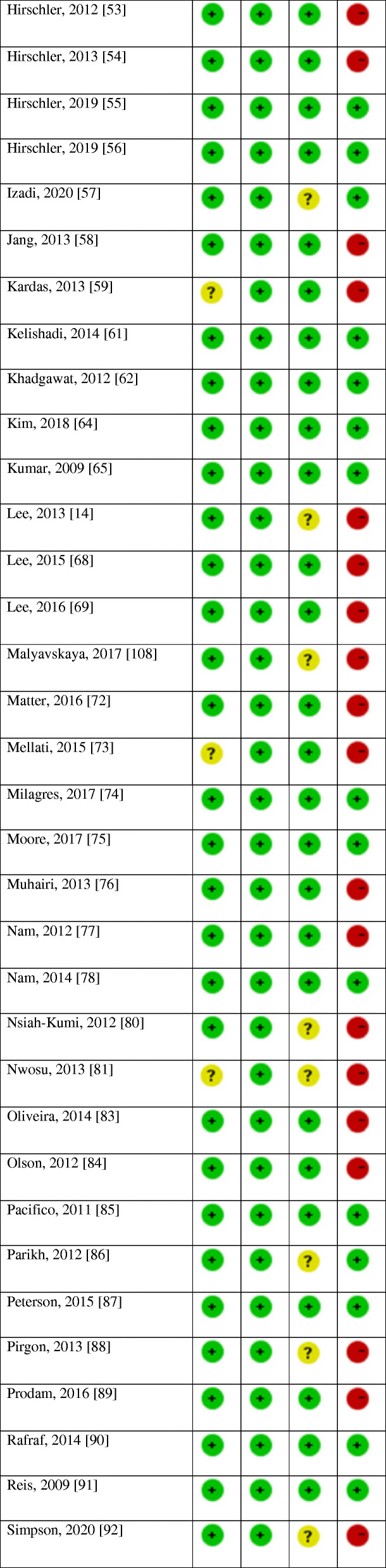

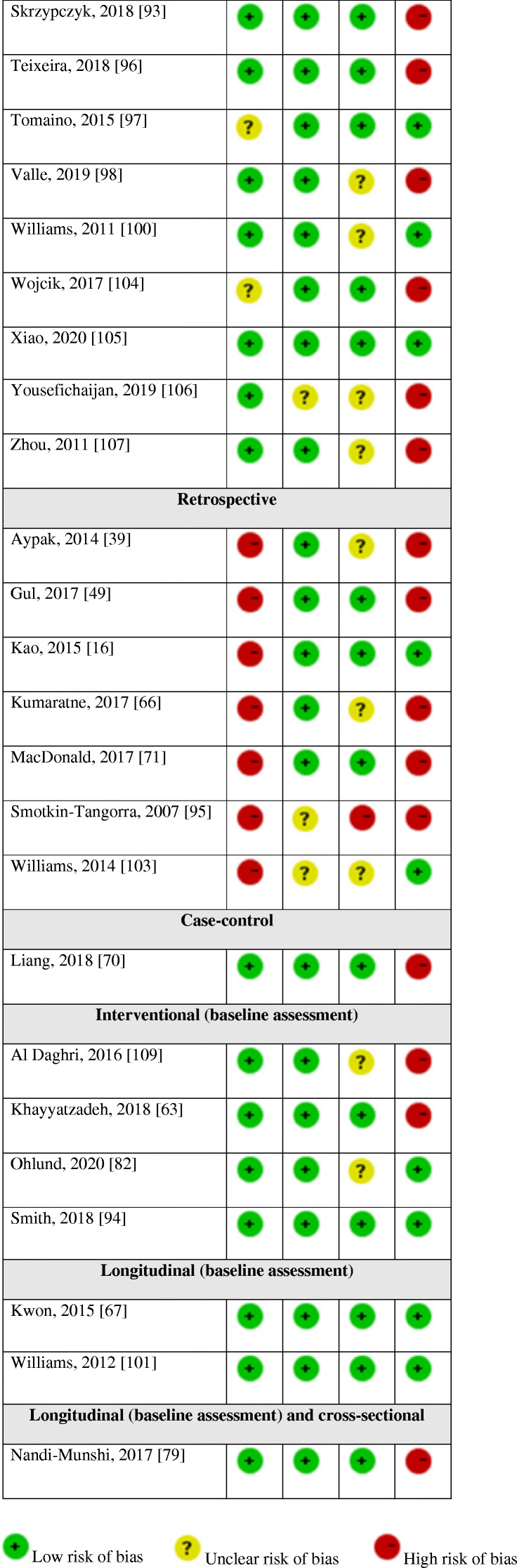

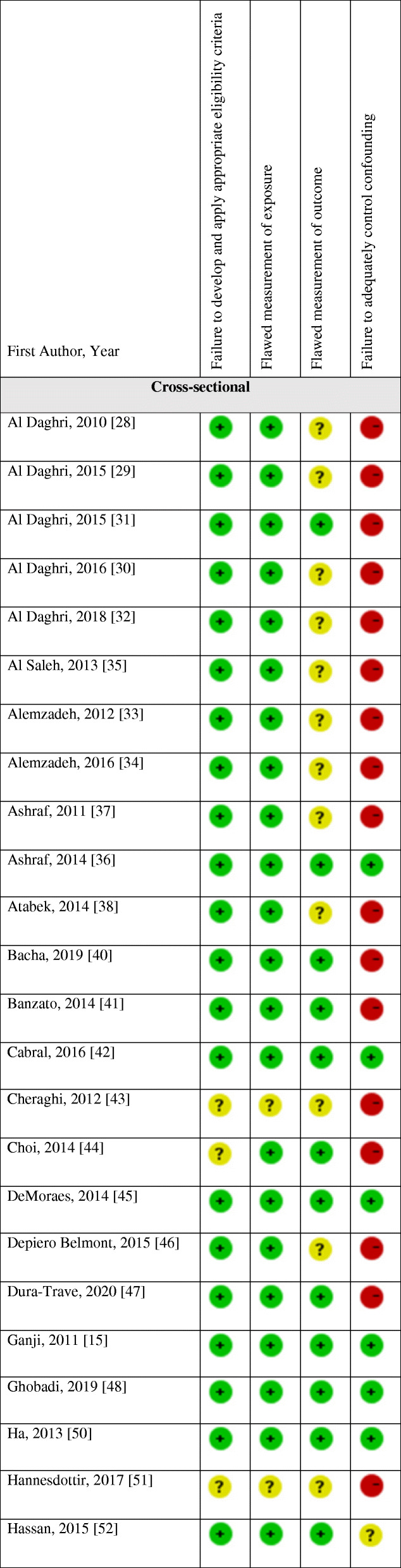
Risk of bias of the non-prospective cohort studies

The assessment of risk of bias of non-prospective cohort studies is presented in Fig. 3. Developing and/or applying appropriate eligibility criteria were flawed in seven studies [16, 39, 49, 66, 71, 95, 103] and were unclear in another eight [43, 44, 51, 59, 73, 81, 97, 104]. Interestingly, seven studies [43, 52, 64, 66, 95, 103, 106] did not describe the measurement of vitamin D, another 30 [14, 16, 29, 32, 37–39, 43, 49, 51, 53, 54, 57, 63, 66, 69, 71, 72, 80–82, 86, 88, 92, 98, 103, 106–109] did not provide a detailed description of BP measurement, and 29 [28, 31, 33–36, 42, 44–46, 48, 50, 52, 55, 56, 58, 59, 61, 67, 68, 76–78, 85, 90, 95, 96, 101, 109] had a high risk of bias for BP measurement. Only 29 studies [15, 16, 36, 42, 45, 48, 50, 55–57, 61, 62, 64, 65, 67, 74, 75, 82, 85–87, 90, 91, 94, 97, 100, 101, 103, 105] provided results adequately adjusted to potential confounders.

## Discussion

Vitamin D deficiency is a global and pressing public health problem even in countries that have an abundance of sunshine throughout the year [110], and in countries where vitamin D supplementation has been implemented for years [8, 111]. Despite the striking lack of data pertaining to children and adolescents worldwide [112], available evidence pinpoints a widespread vitamin D deficiency specifically in this age group [113–118]. This deficiency can be attributed to atmospheric and environmental determinants such as high latitude and air pollution; endogenous characteristics such as genetics, skin pigmentation, obesity, and limited physical activity; and behavioral factors, such as sun avoidance, reduced outdoor activities, and use of sunscreen [119]. Similarly, the prevalence of elevated BP in this age group has been on the rise [2]. Interestingly, factors associated with decreased skin production of vitamin D, including high latitude, industrialization, and dark skin, were also shown to be associated with increased BP levels [120]. Thus, the association between vitamin D and BP gained increasing attention over the last couple of years.

Accumulating evidence ranging from molecular mechanisms to biochemical (physiological) and clinical data suggests that vitamin D deficiency contributes to hypertension and proposes an antihypertensive effect of this vitamin, through vasculo- and renoprotective effects, suppression of the renin-angiotensin-aldosterone system, and effects on calcium homeostasis, among others [120]. Yet, to date, epidemiological and experimental data has not provided conclusive evidence about the association between BP and 25(OH)D concentrations, and available results are inconsistent with neither inverse nor an independent relationship reported [120, 121]. In order to thoroughly investigate the association between vitamin D and BP in children and adolescents, we conducted a systematic review of observational studies. We provided separate analyses for prospective cohort and non-prospective cohort studies (cross-sectional, retrospective, and case-control). We opted this approach since in non-prospective cohort studies, the temporal relationship between exposure and outcome can often not be determined; thus, it is not possible to determine the direction of causality of the reported relationship [122]. In contrast, although observational cohort studies hold a lower position in the evidence rankings compared with randomized controlled trials (RCTs), their longitudinal findings may shed light on the direction of causality [122] and would, therefore, facilitate causal inference [123].

Our search identified three prospective cohort studies that explored the association between childhood 25(OH)D status and BP during later childhood and adolescence. The three studies showed no association between vitamin D and BP. However, due to the limited number of studies and limited sample size, the conclusions that can be drawn from this analysis are considerably limited, and the clinically relevant information provided is not novel.

The vast majority of the studies identified in our review were non-prospective cohort, which mainly did not show a consistent inverse association between 25(OH)D and SBP or DBP levels, or hypertensive status. Possible reasons for the inconsistency in the findings of these studies might relate to the different methodologies used to evaluate vitamin D status and BP, as well as the flawed evaluation of the latter in some studies, the wide difference in the sample size, seasonality, and difference in maturity (Tanner stage) of the participants, in addition to other physiological and environmental factors [6, 11, 12, 25, 124, 125].

In specific, for the majority of these studies, BP evaluation was mainly based on the average of two readings. This method yields a crude estimation of the average BP level and may be subject to artifacts such as regression to the mean. Recent data among children suggest that the first three measurements differ significantly from the average BP, while the fourth till tenth readings do not, and suggest that in children, the fourth BP reading might be used as a reliable approximation, and BP measurement might be improved by including ten measurements [124]. Only two studies [41, 93] adopted ambulatory blood pressure monitoring (ABPM), which is a superior technique [125] and is considered the gold standard of BP measurements in children and adolescents, as it precisely characterizes changes in BP throughout daily activities, correlates more with target organ damage [126, 127]. Benzato et al. [41] evaluated the relationship between 24-h BP patterns and vitamin D levels in 32 obese children and showed that low levels of vitamin D were associated with a higher BP burden, especially at night. In contrast, Skrzypczyk et al. [93] evaluated 49 children with arterial hypertension and found that vitamin D status did not correlate with office BP nor ABPM parameters except for heart rate, suggesting negative influence of vitamin D on arterial wall, which requires further investigations. Up-till-now, ABPM and other reliable estimates of average BP, such as repeated measurements, were rarely used in large epidemiological and clinical studies in children and even in adults [120].

The lack of a significant association between vitamin D status and BP might be attributable to the recruitment of normotensive participants in the vast majority of the studies [121]. Yet, even in studies investigating vitamin D levels across groups of BP status, the results were inconsistent. And, while the majority these studies did not find a difference in vitamin D level among normotensive participants and those with high BP, four found a lower vitamin D level in participants with elevated BP compared with their counterparts. Further investigating the reason behind these mixed results through prospective cohort studies is warranted, especially that Reis et al. [91] who used data from a large sample from NHANES 2001-2004 demonstrated a lower mean vitamin D among subject with high BP. These same inconsistent findings were reported in the adult population [120].

Through a systematic review of the literature investigating the pediatric population, Abboud [24] showed no effect of vitamin D supplementation on SBP or DBP in RCTs, and only a significant small decrease in DBP in non-randomized trials. The quality of evidence of this analysis ranged between low and moderate. Yet, the author acknowledged the small number of included studies, and the fact that all participants were normotensive at baseline, which might mitigate any effect of vitamin D on reducing BP. Similarly, Hauger et al. [128], through a systematic review of RCTs, found no effect of vitamin D supplementation on cardiometabolic health in childhood and adolescence, except for a beneficial impact of increasing 25(OH)D above 70 nmol/L on insulin resistance only in obese subjects, which still needs to be confirmed in adequately powered, high-quality RCTs. The authors acknowledged the low risk of bias in the included studies, but advised caution when interpreting their results as they are based on a relatively limited number of available RCTs. These results are in line with the findings reported among adults showing that vitamin D supplementation did not affect cardiometabolic outcomes, including BP [129–131]. Only, Witham et al. [132], through a systematic review of the literature and a meta-analysis, showed a small but significant fall in DBP with vitamin D supplementation only among adults with elevated BP, whereas no reduction in BP was observed in people who were normotensive at baseline. Accordingly, while observational studies might show associations between low 25(OH)D concentrations and elevated BP, interventional studies have not confirmed that optimizing vitamin D status through supplementation has antihypertensive effects. It could be deduced that associations between 25(OH)D and BP are not causal. This was also suggested by a large systematic review and meta-analysis on the relationship between vitamin D status and a wide range of acute and chronic health disorders [133].

Finally, it is always worthy to note that when discussing the effects of vitamin D on BP, one must consider the extremely rare yet relevant issue of toxicity with mega doses of vitamin D and associated-hypercalcemia [134], which might lead to reversible hypertension [135]. Data from Tomaino et al. [97] indicate a U-shaped relationship between SBP and 25(OH)D, and inverse J-shaped relationships between DBP and MAP with serum 25(OH)D status.

## Strengths and limitations

To our knowledge, this is the first review to systematically assess the association between vitamin D status and BP in children and adolescents, according to a predefined protocol, and following standard methods for reporting systematic reviews [26]. We searched multiple databases and did not set limits to publication language or time, to increase the comprehensiveness of our search. In addition, we critically appraised included studies. Yet, one limitation of our work relates to the quality of the included records. We included observational studies, only three of them were prospective cohort. The remaining included studies were non-prospective cohort (cross-sectional, retrospective, case-control), among which more than half did not adequately control for significant potential confounders. Although the majority of the studies assessed serum concentration of 25(OH)D which is considered the best determinant of vitamin D status [136], few studies adopted rigorous methodology for measuring BP, such as the use of random-zero sphygmomanometers, multiple readings, or automated measurement [132], which may lead to a suboptimal assessment of BP. The quality of evidence of our review is limited by the variable study quality, significant heterogeneity of outcome assessment methods, and study populations. All of which limit our ability to draw a solid conclusion regarding the relationship between vitamin D status and BP. Accordingly, our results suggest of a lack of association, which are certainly not robust enough and should be interpreted with caution.

## Conclusions

Accumulating evidence, ranging from physiological mechanisms, to epidemiological data suggests a link between vitamin D deficiency and elevated BP. Yet, conclusive evidence on the antihypertensive effects of vitamin D remains lacking. The results on the relationship between vitamin D status and BP in children and adolescents varied between the studies, and mainly pointed towards lack of association. In order to provide a clear-cut answer on the antihypertensive effects of vitamin D, future work should assess the effect of vitamin D on BP reduction in hypertensive patients through powered, high-quality and long-term RCTs, and should be followed by larger-scale studies examining the impact of vitamin D on hard outcomes, including cardiovascular events and death. If the association proves to be causal, optimizing vitamin D status through supplementation in the pediatric population could slow the rising BP prevalence in this population group. In light of the widespread vitamin D deficiency, and the various health benefits of this vitamin on the musculoskeletal [137], immune [138], neurological [139], and cardiovascular [140] systems, optimizing vitamin D status in the pediatric population remain needed.

## Supplementary Information

**Additional file 1.**

**Additional file 2.**

**Additional file 3.**

## Data Availability

Not applicable.

## Abbreviations

25(OH)D: Hydroxyvitamin D
ABPM: Ambulatory blood pressure monitoring
ALSPAC: Avon longitudinal study of parents and children
BMI: Body mass index
BP: Blood pressure
CHS: Children’s health study
CVD: Cardiovascular disease
DBP: Diastolic blood pressure
MAP: Mean arterial pressure
MeSH: Medical subject headings
NHANES: National Health and Nutrition Examination Survey
PRISMA: Preferred Reporting Items for Systematic Reviews and Meta-analyses
PROSPERO: International Prospective Register of Systematic Reviews
RCT: Randomized controlled trials
SBP: Systolic blood pressure
USA: United States of America
