# Supplementary material for: Vitamin D status and blood pressure in children and adolescents: a systematic review of observational studies
Source: Syst Rev. 2021 Feb 22;10:60. doi: 10.1186/s13643-021-01584-x (PMC7898425; doi:10.1186/s13643-021-01584-x)
Supplement: Supplementary file 2 — Additional file 2. [file 13643_2021_1584_MOESM2_ESM.docx]

**Additional File 2: Search Strategy**

**MEDLINE**

Database: Ovid MEDLINE(R) ALL <1946 to January 17, 2020>

Search Strategy:

--------------------------------------------------------------------------------

1 exp Vitamin D/ (57819)

2 (Cholecalciferol* or calciol or HYDROXYCHOLECALCIFEROL* or hydroxyvitamins d or CALCIFEDIOL or 25 hydroxyvitamin d3 or 25-hydroxycholecalciferol or calcidiol or DIHYDROXYCHOLECALCIFEROL* or dihydroxyvitamins d or CALCITRIOL or 1 alpha,25 dihydroxyvitamin d3 or 1 alpha,25-dihydroxycholecalciferol or 1,25-dihydroxyvitamin d3 or ergocalciferol* or Dihydrotachysterol or 25-Hydroxyvitamin D 2).mp. [mp=title, abstract, original title, name of substance word, subject heading word, floating sub-heading word, keyword heading word, organism supplementary concept word, protocol supplementary concept word, rare disease supplementary concept word, unique identifier, synonyms] (38570)

3 (vitamin adj (d or d2 or d3 or d-2 or d-3)).mp. [mp=title, abstract, original title, name of substance word, subject heading word, floating sub-heading word, keyword heading word, organism supplementary concept word, protocol supplementary concept word, rare disease supplementary concept word, unique identifier, synonyms] (75439)

4 Vitamin D Deficiency/ (15265)

5 1 or 2 or 3 or 4 (87829)

6 exp Hypertension/ (250658)

7 hypertensi*.mp. (499579)

8 ((increase or elevat* or high*) adj2 blood adj1 pressure).mp. [mp=title, abstract, original title, name of substance word, subject heading word, floating sub-heading word, keyword heading word, organism supplementary concept word, protocol supplementary concept word, rare disease supplementary concept word, unique identifier, synonyms] (38782)

9 ((systolic or diastolic or arterial) adj3 pressure).mp. [mp=title, abstract, original title, name of substance word, subject heading word, floating sub-heading word, keyword heading word, organism supplementary concept word, protocol supplementary concept word, rare disease supplementary concept word, unique identifier, synonyms] (179307)

10 7 or 8 or 9 (627657)

11 adolescent/ or exp child/ (2958419)

12 (child or children or childhood or youth or teenage* or p?ediatric*).mp. [mp=title, abstract, original title, name of substance word, subject heading word, floating sub-heading word, keyword heading word, organism supplementary concept word, protocol supplementary concept word, rare disease supplementary concept word, unique identifier, synonyms] (2477749)

13 (adolescen* or (school adj age) or youngster*).mp. [mp=title, abstract, original title, name of substance word, subject heading word, floating sub-heading word, keyword heading word, organism supplementary concept word, protocol supplementary concept word, rare disease supplementary concept word, unique identifier, synonyms] (2066781)

14 11 or 12 or 13 (3499936)

15 5 and 10 and 14 (324)

16 5 and 10 (2408)

17 limit 16 to "all child (0 to 18 years)" (259)

18 15 or 17 (349)

19 (pediatric? or child or children or adolescen* or youth).jw. (542548)

20 16 and 19 (58)

21 18 or 20 (353)

**EMBASE**

Database: Embase Classic <1947 to 1973>, Embase <1974 to 2020 January 17>

Search Strategy:

--------------------------------------------------------------------------------

1 exp vitamin D/ (140645)

2 exp vitamin D deficiency/ (28964)

3 (Cholecalciferol* or calciol or HYDROXYCHOLECALCIFEROL* or hydroxyvitamins d or CALCIFEDIOL or 25 hydroxyvitamin d3 or 25-hydroxycholecalciferol or calcidiol or DIHYDROXYCHOLECALCIFEROL* or dihydroxyvitamins d or CALCITRIOL or 1 alpha,25 dihydroxyvitamin d3 or 1 alpha,25-dihydroxycholecalciferol or 1,25-dihydroxyvitamin d3 or ergocalciferol* or Dihydrotachysterol or 25-Hydroxyvitamin D 2).mp. (52332)

4 (vitamin adj (d or d2 or d3 or d-2 or d-3)).mp. (130950)

5 1 or 2 or 3 or 4 (162755)

6 exp hypertension/ (733633)

7 hypertensi*.mp. (938420)

8 ((increase or elevat* or high*) adj2 blood adj1 pressure).mp. (58276)

9 ((systolic or diastolic or arterial) adj3 pressure).mp. (355528)

10 6 or 7 or 8 or 9 (1221108)

11 child/ (1769502)

12 exp adolescent/ (1484727)

13 (child or children or childhood or youth or teenage* or p?ediatric*).mp. (2867838)

14 (adolescen* or (school adj age) or youngster*).mp. (1596691)

15 11 or 12 or 13 or 14 (3542372)

16 5 and 10 and 15 (1398)

17 5 and 10 (10579)

18 limit 17 to child <unspecified age> (646)

19 16 or 18 (1398)

**CINAHL**

| **#** | **Query** | **Limiters/Expanders** | **Results** |
| --- | --- | --- | --- |
| S17 | S14 OR S16 | Expanders - Apply equivalent subjects  Search modes - Boolean/Phrase | 83 |
| S16 | S5 AND S9 | Expanders - Apply equivalent subjects  Narrow by SubjectAge0: - all child  Search modes - Boolean/Phrase | 71 |
| S15 | S5 AND S9 | Expanders - Apply equivalent subjects  Search modes - Boolean/Phrase | 463 |
| S14 | S5 AND S9 AND S13 | Expanders - Apply equivalent subjects  Search modes - Boolean/Phrase | 83 |
| S13 | S10 OR S11 OR S12 | Expanders - Apply equivalent subjects  Search modes - Boolean/Phrase | 961,298 |
| S12 | Ti (youngster or school age or adolescen* or paediatric* or pediatric* or teenage* or youth or child*) or ab (youngster or school age or adolescen* or paediatric* or pediatric* or teenage* or youth or child*) | Expanders - Apply equivalent subjects  Search modes - Boolean/Phrase | 398,135 |
| S11 | (MH "Adolescence+") | Expanders - Apply equivalent subjects  Search modes - Boolean/Phrase | 477,443 |
| S10 | (MH "Child+") | Expanders - Apply equivalent subjects  Search modes - Boolean/Phrase | 596,921 |
| S9 | S6 OR S7 OR S8 | Expanders - Apply equivalent subjects  Search modes - Boolean/Phrase | 81,207 |
| S8 | ti (high or elevated or increase* or systolic or diastolic or arterial) n3 pressure* or ab (high or elevated or increase* or systolic or diastolic or arterial) n3 pressure* | Expanders - Apply equivalent subjects  Search modes - Boolean/Phrase | 5,573 |
| S7 | ti hypertensi* or ab hypertensi* | Expanders - Apply equivalent subjects  Search modes - Boolean/Phrase | 35,745 |
| S6 | (MH "Hypertension+") | Expanders - Apply equivalent subjects  Search modes - Boolean/Phrase | 69,915 |
| S5 | S1 OR S2 OR S3 OR S4 | Expanders - Apply equivalent subjects  Search modes - Boolean/Phrase | 20,348 |
| S4 | Ti (vitamin d or vitamin d2 or vitamin d3 or vitamin d 3 or vitamin d 2) or ab (vitamin d or vitamin d2 or vitamin d3 or vitamin d 3 or vitamin d 2) | Expanders - Apply equivalent subjects  Search modes - Boolean/Phrase | 10,864 |
| S3 | Ti (alpha,25-dihydroxycholecalciferol or ergocalciferol* or Dihydrotachysterol or dihydroxyvitamin d or dihydroxyvitamins d or 1 alpha,25 dihydroxyvitamin d3 or 1 alpha,25-dihydroxycholecalciferol or 1,25-dihydroxyvitamin d3 or 25-Hydroxyvitamin D 2) or ab (alpha,25-dihydroxycholecalciferol or ergocalciferol* or Dihydrotachysterol or dihydroxyvitamin d or dihydroxyvitamins d or 1 alpha,25 dihydroxyvitamin d3 or 1 alpha,25-dihydroxycholecalciferol or 1,25-dihydroxyvitamin d3 or 25-Hydroxyvitamin D 2) | Expanders - Apply equivalent subjects  Search modes - Boolean/Phrase | 101 |
| S2 | Ti (Cholecalciferol* or calciol or HYDROXYCHOLECALCIFEROL* or CALCIFEDIOL or 25-hydroxycholecalciferol or calcidiol or DIHYDROXYCHOLECALCIFEROL* or CALCITRIOL) or ab (Cholecalciferol* or calciol or HYDROXYCHOLECALCIFEROL* or CALCIFEDIOL or 25-hydroxycholecalciferol or calcidiol or DIHYDROXYCHOLECALCIFEROL* or CALCITRIOL) | Expanders - Apply equivalent subjects  Search modes - Boolean/Phrase | 360 |
| S1 | (MH "Vitamin D+") OR (MH "Vitamin D Deficiency+") | Expanders - Apply equivalent subjects  Search modes - Boolean/Phrase | 18,743 |

**PUBMED**

| Search | Query | Items found |
| --- | --- | --- |
| #29 | Search (#7 and #17 and #28) | 3648 |
| #28 | Search (#18 or #19 or #20 or #21 or #22 or #23 or #24 or #25 or #26 or #27) | 3454758 |
| #27 | Search youngster*[tiab] | 2476 |
| #26 | Search school age[tiab] | 13013 |
| #25 | Search adolescen*[tiab] | 286605 |
| #24 | Search paediatric*[tiab] | 64591 |
| #23 | Search pediatric*[tiab] | 289152 |
| #22 | Search teenage*[tiab] | 20898 |
| #21 | Search youth[tiab] | 68591 |
| #20 | Search ((child[tiab] or children[tiab] or childhood[tiab])) | 1333474 |
| #19 | Search child[mesh] | 1872154 |
| #18 | Search adolescent[mesh] | 1983762 |
| #17 | Search (# 8 or #9 or #10 or #11 or #12 or #13 or #14 or #15 or #16) | 4646659 |
| #16 | Search (hypertension[tiab] or hypertensive[tiab] or hypertensives[tiab]) | 426502 |
| #15 | Search arterial pressure[tiab] | 58738 |
| #14 | Search diastolic pressure[tiab] | 13643 |
| #13 | Search systolic pressure[tiab] | 13954 |
| #12 | Search increase blood pressure*[tiab] | 760 |
| #11 | Search elevated blood pressure[tiab] | 6289 |
| #10 | Search ((systolic blood pressure*[tiab] or diastolic blood pressure*[tiab] or arterial blood pressure*[tiab])) | 98847 |
| #9 | Search high blood pressure*[tiab] | 14672 |
| #8 | Search hypertension[mesh] | 250320 |
| #7 | Search (#1 or #2 or #3 or #4 or #5 or #6) | 87699 |
| #6 | Search (vitamin d[tiab] or vitamin d2[tiab] or vitamin d3[tiab] or vitamin d 3[tiab] or vitamin d 2[tiab]) | 66554 |
| #5 | Search (dihydroxyvitamin d[tw] or dihydroxyvitamins d[tw] or 1 alpha,25 dihydroxyvitamin d3[tw] or 1 alpha,25-dihydroxycholecalciferol[tw] or 1,25-dihydroxyvitamin d3[tw] or 25-Hydroxyvitamin D 2[tw]) | 12196 |
| #4 | Search 25 hydroxyvitamin d3 [tw] | 2989 |
| #3 | Search (Cholecalciferol*[tw] or calciol[tw] or HYDROXYCHOLECALCIFEROL*[tw] or CALCIFEDIOL[tw] or 25-hydroxycholecalciferol[tw] or calcidiol[tw] or DIHYDROXYCHOLECALCIFEROL*[tw] or CALCITRIOL[tw] or alpha,25-dihydroxycholecalciferol[tw] or ergocalciferol*[tw] or Dihydrotachysterol[tw]) | 36629 |
| #2 | Search vitamin D deficiency [mesh:noexp] | 15220 |
| #1 | Search vitamin D [mesh] | 57729 |

**Cochrane Library**

#1 MeSH descriptor: [Vitamin D] explode all trees 4778

#2 MeSH descriptor: [Vitamin D Deficiency] explode all trees 1269

#3 (Cholecalciferol* or calciol or HYDROXYCHOLECALCIFEROL* or CALCIFEDIOL):ti,ab,kw 3039

#4 (calcidiol or DIHYDROXYCHOLECALCIFEROL* or CALCITRIOL):ti,ab,kw 1897

#5 (ergocalciferol* or Dihydrotachysterol or dihydroxyvitamin d or dihydroxyvitamins d or 1 alpha,25 dihydroxyvitamin d3):ti,ab,kw 1826

#6 (vitamin d):ti,ab,kw 12995

#7 (vitamin d2 or vitamin d 3):ti,ab,kw 7528

#8 #1 or #2 or #3 or #4 or #5 or #6 or #7 14586

#9 MeSH descriptor: [Hypertension] explode all trees 17001

#10 (hypertensi* or (high or elevated or increase* or systolic or diastolic or arterial) near/3 pressure*):ti,ab,kw 568810

#11 #9 or #10 568810

#12 MeSH descriptor: [Child] explode all trees 1209

#13 MeSH descriptor: [Adolescent] explode all trees 102003

#14 (youngster or school age or adolescen* or paediatric* or pediatric* or teenage* or youth or child*):ti,ab,kw 243608

#15 #12 or #13 or #14 243608

#16 #8 and #11 and #15 1559

1525 Trials matching "#16 - #8 and #11 and #15"

**ClinicalTrial.gov**

Hypertension | Vitamin D | Child

Applied filer: Child (birth-17)
