# Supplementary material for: Vitamin D status and blood pressure in children and adolescents: a systematic review of observational studies
Source: Syst Rev. 2021 Feb 22;10:60. doi: 10.1186/s13643-021-01584-x (PMC7898425; doi:10.1186/s13643-021-01584-x)
Supplement: Supplementary file 3 — Additional file 3. [file 13643_2021_1584_MOESM3_ESM.docx]

**Additional File 3: Detailed numerical results of the studies (classified by study design, exposure and level of statistical adjustment)**

| **First Author, Year- Country** | **Outcomes Evaluated and Evaluation Method** | **Adjusted variables for statistical analysis** | **Results** | **Key Findings** |
| --- | --- | --- | --- | --- |
| **Cross-sectional** | | | | |
| Al Daghri, 2010- Riyadh, Saudi Arabia [28] | Serum 25(OH)D: ELISA SBP and DBP: average of 2 readings (not detailed) | None | Significant inverse correlation between 25(OH)D and SBP [All: r=-0.24 (p<001); Girls: -0.37 (p<0.01); Boys: -0.18 (NS)] Significant inverse correlation between 25(OH)D and DBP [All: r=-0.22 (p<0.05); Girls: -0.29 (p:<0.01); Boys: -0.19 (NS)] | Inverse correlation between vitamin D with SBP and DBP in the total sample and in girls, but not in boys |
| Al Daghri, 2015- Riyadh, Saudi Arabia [29] | Serum 25(OH)D: COBAS e-411 automated analyzer SBP and DBP: NR | None | No significant correlation between 25(OH)D and SBP [All: r= -0.03(NS); Girls: r=0.02(NS); Boys: r=-0.28(p< 0.01)] Significant inverse correlation between 25(OH)D and DBP [All: r= -0.14(p<0.05); Girls: r= -0.16(NS); Boys: r= -0.09(NS)] | ● Inverse correlation between vitamin D with SBP in boys only ● Inverse correlation between vitamin D with DBP in the total sample |
| Al Daghri, 2015- Riyadh, Saudi Arabia [31] | Serum 25(OH)D: COBAS e-411 automated analyzer SBP and DBP: average of 2 readings, 15 minutes apart, by a standardized mercury sphygmomanometer | None | Inverse correlation between 25(OH)D and SBP [boys: -0.1 (p<0.01); girls: 0.02 (NS)]  Inverse correlation between 25(OH)D and DBP [boys: -0.09 (p<0.01); girls: 0.05 (NS)] | Inverse correlation between vitamin D with SBP and DBP in boys only |
| Al Daghri, 2016- Riyadh, Saudi Arabia [30] | Serum 25(OH)D: COBAS e-411 automated analyzer SBP and DBP: NR | None | NS Inverse correlation between serum log 25(OH)D and SBP (All:r= -0.03 ; boys: r=-0.16(p<0.01); Girls: r=0.01(NS) NS inverse correlation between serum log 25(OH)D and DBP(All: r=-0.02; boys: r=-0.06(p<0.05); Girls: r=0.02(NS) | Inverse correlation between vitamin D with SBP and DBP in boys only |
| Choi, 2014- South Korea [44] | Serum 25(OH)D: radioimmunoassay SBP and DBP: average of 2 readings, at rest, using a oscillometric device with appropriate cuff size | None | Boys: NS correlation between 25(OH)D and SBP (r= 0.04; p: NS). NS correlation between 25(OH)D and DBP (r= 0.038; p: NS) Girls: NS correlation between 25(OH)D and SBP.NS correlation between 25(OH)D and DBP (r=-0.024;p: NS) (r=0.09; p:NS) | No correlation between Vitamin D with SBP and DBP |
| Hassan, 2015- Egypt [52] | Serum 25(OH) D: NR SBP and DBP: average of 2 readings by a mercury sphygmomanometer after a rest of 20 minutes | None | Total sample:  NS association between 25(OH)D levels and SBP (R=0.187 p=0.136) NS association between 25(OH)D levels and DBP (R= 0.006 p=0.963)  Obese: NS association between 25(OH)D levels and SBP (R=0.187; p=0.136) NS association between 25(OH)D levels and DBP (R=0.006; p=0.963) | No association between vitamin D with SBP and DBP in the total sample, and in obese participants |
| Kardas, 2013- Turkey [59] | Plasma 25(OH)D: HPLC SBP and DBP: average of 2 readings by a mercury sphygmomanometer, after a 20 minutes rest | None | No significant correlation between serum 25(OH)D and SBP among obese (r=0.33, p=0.796) and non obese (r=0.13, p=0.926)groups. No significant correlation between serum 25(OH)D and DBP among obese (r=-0.66, p=0.608) and non obese (r=-0.252, p=0.75) groups Significant association between serum 25(OH)D and SBP (r=-0.190, p=0.043) in the total population Significant inverese correlation between serum 25(OH)D and DBP (r=-0.392, p=000) in the total population | Inverse correlation between Vitamin D with SBP and DBP in the total sample; no longer significant when assessed among obese and non-obese participants |
| Pirgon, 2013-Turkey [88] | Serum 25(OH)D: automated chemiluminescence immunoassay SBP and DBP: mercury-gravity manometer and a cuff appropriate for body size, in a sitting position, after rest for at least 5 min | None | NS positive correlation between 25(OH)D and SBP [without NAFLD: r: 0.051; p= 0.842; with NAFLD: r: 0.29; p=0.529] NS positive correlation between 25(OH)D and DBP [without NAFLD: r: 0.164; p= 0.516; with NAFLD: r: 0.047; p= 0.920] | No correlation between Vitamin D with SBP and DBP in participants with and without NAFLD |
| Prodam, 2016- Novara area, Italy [89] | Serum 25(OH)D: direct competitive chemiluminescent immunoassay SBP and DBP: average of 3 measurements on the left arm, after a 15-minute rest in the supine position and prior to other physical evaluations, using a standard mercury sphygmomanometer | None | Significant negative correlation between Log25OHD and LogSBP: r= -0.119; p=0.005 NS negartive correlation between Log25OHD and LogDBP: r= -0.069; p=0.102 | Inverse correlation between Vitamin D and SBP only |
| Simpson, 2020-Connecticut, USA [92] | Total serum 25-OHD and 1,25(OH)2D: radioimmunoassay Calculated free 25(OH)D: calculated using serum vitamin D binding protein and albumin concentrations, and their reported dissociation constants  Genotype-specific free 25(OH)D: calculated using 25(OH)D/DBP dissociation constants specific for each individual’s haplotype Direct measured free 25(OH)D: ELISA SBP and DBP: NR | None | Significant negative correlation between Total 25(OH)D and SBP (r= -0.16; p<0.05) NS correlation between Total 25(OH)D and DBP (r = -0.5; p>0.05) Significant negative correlation between calculated free 25(OH)D and SBP (r= -0.22; p<0.01) NS correlation between calculated free 25(OH)D and DBP (r = -0.14; p>0.05) NS correlation of genotype-specific calculated free 25(OH)D with SBP (r = -0.05; p>0.05) NS correlation of genotype-specific calculated free 25(OH)D with DBP (r = 0.02; p>0.05) Significant negative correlation between directly measured free 25(OH)D and SBP (r= -0.23; p<0.01) NS correlation between directly measured free 25(OH)D and DBP (r = -0.1; p>0.05) | Inverse correlation between Vitamin D with SBP only |
| Skrzypczyk, 2018- Poland [93] | 25(OH)D: chemiluminescence Peripheral BP: using oscillometric device 24-hour BP: using a SUNTECH OSCAR 2 device and interpreted according to the American Heart Association guidelines. Monitors were programmed to measure BP every 15 minutes from 6 AM to 10 PM and every 30 minutes from 10 PM to 6 AM SBP, DBP and MAP: measured during 24 hours | None | NS correlation between 25(OH)D and BP  Office BP: SBP [mm Hg] r = –0.14 p = 0.33; DBP [mm Hg] r = –0.19 p = 0.18  Ambulatory blood pressure monitoring SBP during 24h [mm Hg] r = –0.14 p = 0.34; DBP during 24h [mm Hg] r = –0.17 p = 0.25 MAP during 24h [mm Hg] r = –0.15 p = 0.29; MAP during 24h Z-score r = –0.23 p = 0.11 | No correlation between Vitamin D with SBP and DBP |
| Zhou, 2011- USA [107] | Serum 25(OH)D: chemiluminescent assay SBP and DBP: NR | Age | Negative correlation between 25(OH)D and SBP (r= -0.261; p=0.038) NS correlation between 25(OH)D and DBP (r=-0.175; p= NS)  SBP significantly higher in 25(OH)D <25 nmol/l group (ΔSBP= 9.24; p=0.03); <37.5 nmol/l (ΔSBP= 8.50; p=0.008); <50 nmol/l (ΔSBP= 7.79; p=0.007); ≤62.5 nmol/l (ΔSBP= 8.82; p=0.003) compared with the 25(OH)D >62.5 nmol/l group NS difference for DBP (data not shown)  The hockey stick model analysis found some evidence of a threshold model in SBP, with a breakpoint at 25(OH)D =26.9 ng/mL. The slope on the right side of this breakpoint is statistically significantly different from 0. The slope on the left side is not significantly different from 0. The model as a whole explains 17% of the variance in SBP | ● Negative correlation between Vitamin D with SBP only ● Higher SBP with Vitamin D deficiency |
| Al Saleh, 2013-Riyadh, Saudi Arabia [35] | Serum 1,25(OH)2D: ELISA  SBP and DBP: average of 2 readings (not detailed) | BMI; Gender | Inverse correlation between 1,25-(OH) 2 D and SBP (r= − 0.50; p=0.01) and NS with DBP (r= − 0.37; p=0.05) Inverse association between 1,25(OH)D and SBP[ R= -0.46 (p=0.008)] Inverse association between 1,25(OH)D and DBP[R= -0.3 (p=0.33)(NS)] | Inverse association between vitamin D with SBP only |
| Ashraf, 2011- Birmingham, USA [37] | Serum 25(OH)D: liquid chromatography-tandem mass spectrometry SBP and DBP: automated BP cuff (not detailed) | BMI; Race | NS correlation between serum 25(OH)D and SBP [r=-0.07 (p: 0.53)] and DBP [r=0.04 (p: 0.71)] | No correlation between Vitamin D with SBP and DBP |
| Olson, 2012- North Texas, USA [84] | 25(OH)D: chemiluminescent immunoassay SBP and DBP: average of up to 3 measures, by dinamap procare monitor, at rest | BMI z-scores; Age | NS inverse correlation between serum 25(OH)D and SBP (r=-0.07; p=0.14) NS inverse correlation between serum 25(OH)D and DBP (r=-0.01; p=0.86) | No correlation between Vitamin D with SBP and DBP |
| Ashraf, 2014- Alabama, USA [36] | Serum 25(OH)D: liquid chromatography mass spectrometry Free 25(OH)D and bioavailable 25(OH)D: calculated using published formulas SBP ad DBP: average of 2 readings, after a 5-minute rest, using the auscultatory method, in supine position | Age; Percent body fat; Race; Fasting insulin; Height | NS correlation between vitamin D and BP Total 25(OH)D SBP (r= -0.04; p=0.81); DBP (r= 0.21 ; p=0.21) Free 25(OH)D SBP (r= -0.2; p= 0.24); DBP (r= 0.14; p=0.23) Bioavailable 25(OH)D SBP (r= -0.18; p=0.27); DBP (r= 0.17; p=0.33) | No correlation between Vitamin D with SBP and DBP |
| Atabek, 2014- Konya, Turkey [38] | Serum 25(OH)D: mass spectrometry  Vitamin D status groups: Deficient:<50 nmol/L SBP and DBP: after a rest ≥10 minutes, using a standard mercury sphygmomanometer | None | NS association between vitamin D status and SBP [deficient: 119.43±17.44, non-deficient: 116.21±18.12; p:0.19] and DBP [deficient: 73.13±13.14, non-deficient: 74.08±13.24; p: 0.61] Inverse correlation between 25(OH)D and SBP [r=-0.169; p=0.016] | ● No differences in SBP and DBP across Vitamin D status groups ● Inverse correlation between Vitamin D with SBP only |
| Al Daghri, 2018- Riyadh, Saudi Arabia [32] | Serum 25(OH)D: COBAS e-411 automated analyzer Vitamin D status groups: Deficient: 50-75 nmol/L; Insufficient: <50 nmol/L; Sufficient: ≥75nmol/L SBP and DBP: NR | None | NS association in SBP according to vitamin D status: [deficient group: SBP: 119.1 ± 14.5 ; insufficient: 119.9 ± 13.2; sufficient: 117.2 ± 15.4 (p=0.23 )] Significant association in DBP according to 25(OH)D status: [deficient group: DBP: 72.2 ± 10.1 ; insufficient: 69.9 ± 9.4 ; Sufficient: 68.2 ± 9.6 (p=0.005)] | ● No difference in SBP across Vitamin D status groups ● Lower DBP in Vitamin D sufficiency |
| Alemzadeh, 2012-Wisconsin, USA [33] | Serum 25(OH)D: radioimmunoassay Vitamin D status groups: Deficient: <50 nmol/L; Sufficient: ≥50nmol/L SBP and DBP: average of 2 readings, in sitting position | None | NS assocation in SBP according to 25(OH)D status: [mean(SD): Deficient group:126.7(13.5); sufficient group:129.6(13.6), p(NS)] NS association in DBP according to 25(OH)D status: [mean (SD): deficient group:67.8(10.1); Sufficient group: 68.3(8.9) p(NS)] NS correlation between 25(OH)D and SBP (r=0.04; p: NS) and DBP (r=0.08; p: NS) | ● No differences in SBP and DBP across Vitamin D status groups ● No correlation between Vitamin D with SBP and DBP |
| Alemzadeh, 2016-Wisconsin, USA [34] | Serum 25(OH)D: radioimmunoassay  Vitamin D status groups: Deficient: <50 nmol/L; Insufficient: 50-74.9 nmol/L; Sufficient: ≥75 nmol/L SBP and DBP: average of 2 readings, in sitting position | None | NS association between serum 25(OH)D and SBP [All:129(12.2);sufficient:128.9(15.1); insufficient: 30.0(12.7) ;deficient:128.5(11.0); p(NS)] NS association between serum 25(OH)D and DBP [All:69.1(9.5);sufficient:68.2(11.2); insufficient: 69.9(7.9) ;deficient:69.3(10.1); p(NS)] | No differences in SBP and DBP across Vitamin D status groups |
| Cheraghi, 2012- Kansas, USA [43] | Serum 25(OH)D: NR Vitamin D status groups: Deficient: < 49.92 nmol/L; Sufficient: ≥ 49.92 nmol/L SBP: over the right arm, in sitting position, using a dinamap monitor | None | NS association SBP Z score 0.7±0.9 in deficient vs. 0.7±0.9 in sufficient  NS association between 25(OH)D and SBP [mean(SD) 25(OH)D 58.6(12.7) in SBP ≥95th Percentile vs.65.3(25.7) nmol/l in <95th Percentile] | ● No difference in SBP across Vitamin D status groups ● No difference in Vitamin D level among those with normal and high SBP |
| Dura-Trave, 2020- Pamplona, Spain [47] | Plasma 25(OH)D: high-specific chemiluminescence immunoassay Vitamin D status group: Deficient: <50 nmol/L; hypovitaminosis D: <75 nmol/L SBP and DBP: lowest of 3 measurements, in the right arm, in supine position, using a digital BP monitor Arterial HTN: SBP and/or DBP ≥95th p for age, sex, and height, according to the American reference charts (SBP >130 or DBP >85 mm Hg) | None | Higher BP with Vitamin D deficiency  SBP: deficieny: 132.3(10.1); Insufficiency: 127.9(11.5); Sufficiency: 125.2(9.5); p=0.003 DBP: deficieny: 76.8(9.8); Insufficiency: 73.3(9.3); Sufficiency: 73.8(9.4); p=0.036  Higher prevalence of arterial hypertension with hypovitaminosis (deficiency and insufficiency) SBP>130 mmHg: deficieny: 37.8%; Insufficiency: 55.6%; Sufficiency: 37.5%; p=0.038 DBP>85 mmHg: deficiency: 81.1%; Insufficiency: 94.4%; Sufficiency: 75%; p=0.011  Significant negative correlation between 25(OH)D and SBP (r=-0.191; p=0.009) NS correlation between 25(OH)D and DBP (r=-0.067; p=0.363) | ● Higher SBP and DBP with Vitamin D deficiency ● Higher prevalence of arterial HTN with hypovitaminosis D ● Inverse association between vitamin D with SBP only |
| Hannesdottir, 2017- Iceland [51] | Serum 25(OH)D: radioimmuniassay  Vitamin D status groups: Deficient: <50 nmol/L; Sufficient: >50 nmol/L SBP and DBP: average of 3 readings in a standard way using the left arm | None | NS association between Vitamin D and SBP and DBP SBP mean(SD): 94(7) in <37.5 vs. 95(7) in 37.5-50; and 94(6) in deficienct group DBP mean(SD): 55(5) <37.5 vs. 57(5) in 37.5-50; and 56(5) in >sufficient group  SBP: 94(6) in <50 vs. 95(7) in >50 nmol/l; p=0,504; 95%CI: -1,44 to 2,91 DBP: 56(4) in <50 vs. 56 (5) in >50 nmol/l; p=0,335; 95%CI: -2,4 to 0,81  NS association between 25(OH)D and with SBP (r=0.01,p=0,860) NS association between 25(OH)D and DBP (r=0.14, p=0,095) | ● No association/correlation between Vitamin D with SBP and DBP ● No differences in SBP and DBP across Vitamin D status groups |
| Lee, 2016- USA [69] | Plasma total 25(OH)D: in duplicates by Immunodiagnostic Systems enzyme immunoassay SBP and DBP: standard sphygmomanometer in a sitting position with an appropriate size cuff | None | NS assocation between vitamin D and SBP and DBP SBP mean(SD): 126.6(10.9) in non-deficient vs. 127.1(11.7) in deficient DBP mean(SD): 69.1(7.7) in non-deficient vs. 70.5(10.1) in deficient | No association between Vitamin D with SBP and DBP |
| Matter, 2016-KSA [72] | Serum 25(OH)D: radioimmunoassay Vitamin D status: Deficient: <50 nmol/L SBP and DBP: NR | None | NS difference in SBP between defcient 110.5(15.07) and non deficient groups 113.5(12.25) p = 0.76  NS difference in DBP between defcient 81.0(11.78) and non deficient groups 79.88(11.59) for SBP p= 0.06 | No differences in SBP and DBP across Vitamin D status groups |
| Nwosu, 2013-Central New England, USA [81] | Serum 25(OH)D: chemiluminescent immunoassay Vitamin D status groups: Deficient:< 50 nmol/L; Sufficient: >50 nmol/L SBP and DBP: NR | None | NS difference of SBP and DBP between categories of vitamin D SBP: 105.55±9.47 for deficiency vs. 103.88±14.37 for sufficiency; p=0.722 DBP: 65.27±8.34 for deficiency vs. 61.18±9.55 for sufficiency; p=0.228 | No differences in SBP and DBP across Vitamin D status groups |
| Valle, 2019- Rio de Janeiro, Brazil [98] | Serum 25(OH)D: HPLC Vitamin D status group: Deficient: <50 nmol/L SBP and DBP: by an automatic inflation BP monitor (not detailed) High BP: NR | None | NS association between serum vitamin D levels and SBP (r = −0.12 p = 0.24) Significant association between serum vitamin D levels and DBP (r = −0.28p = 0.005)  Higher SBP with vitamin D deficiency (<50 vs. ≥50 nmol/l) SBP: mean(SD): 127.6(15.8) vs. 120(13.7); p=0.007 DBP: mean(SD): 76.3(13.5) vs. 74.5(11.6); p=0.25  Higher prevalence of High Blood pressure with vitamin D deficiency (<50 vs. ≥50 nmol/l): 66.7% vs. 51.6% (p-value not shown) | ● Inverse association between vitamin D with DBP only ● Higher prevalence of elevated BP with vitamin D deficiency ● Higher SBP with vitamin D deficiency |
| Wojcik, 2017- Krakow, Poland [104] | Serum 25(OH)D: HPLC Vitamin D status group: Deficient: <50 nmol/L SBP and DBP: average of 3 measurements, every 3 minutes, using a pneumatic sphygmomanometer Arterial HTN: mean SBP and/or DBP >95th p for age, height and gender | None | NS difference in SBP between vitamin D status group: 125.9(11.1) vs. 115(15); p=0.07 Significantly higher DBP in the deficient group: 78.6(11.5) vs. 59(10.2); p=0.04 Higher prevalence of arterial hypertension in the deficient group: 44% vs. 8.3% (p= NR) | Higher prevalence of arterial HTN and DBP with Vitamin D deficiency; no difference in SBP |
| Kumaratne, 2017- California, USA [66] | Serum 25(OH)D: NR Vitamin D status groups: Deficient: <50 nmol/L; Adequate: ≥50 nmol/L SBP and DBP: NR | None | No significant difference in SBP between vitamin D deficient (117.41 ± 14.1) and vitamin D adequate (116.98 ± 14) p=.8683 in overweight and obese BMI 85% to >99%  No significant difference in SBP between vitamin D deficient (108.95 ± 14.04) and vitamin D adequate (107.6 ± 11.82) p=.6532 in Under and Healthy Weight Subjects, BMI <5% to 84% | No differences in SBP and DBP across Vitamin D status groups, among all weight categories |
| MacDonald, 2017- Alberta, Canada [71] | Serum 25(OH)D: according to standard methodologies Vitamin D status groups: Deficient: <50 nmol/L; Sufficient: >50 nmol/L SBP and DBP: by an automatic BP machine with appropriate cuff size | None | NS association between groups of serum 25(OH)D and SBP deficient: 120(10) and sufficient: 117(13); p=0.08 NS association between groups of serum 25(OH)D and DBP deficient: 67(9) and sufficient: 64(9); p=0.13 | No differences in SBP and DBP across Vitamin D status groups |
| Jang, 2013- South Korea [58] | Serum 25(OH)D: gamma counter with a radioimmunoassay Vitamin D status: Deficient: <50 nmol/L SBP and DBP: average of 2 readings by a mercury sphygmomanometer in a sitting position at rest High BP: >=130/85 mmHg | BMI-z score; Physical activity | NS association between serum 25(OH) D and SBP (unadjusted r=-0.091, p=0.01044; Adjusted r=-0.0547, p=0.3612) NS association between serum 25(OH) D and DBP (unadjusted r=-0.0535, p=0.3415; Adjusted r=-0.0138, p=0.8181)  Significantly higher SBP across vitamin D groups: SBP mean(SD): 108.0(9.3) in deficient group vs. 105.5(9.7) in sufficient group; p=0.0199 NS difference in DBP: DBP mean(SD): 70.0(8.2) in deficient vs. 68.8(8.7) in sufficient; p=0.2109  NS difference in prevalence of High BP (>=130/85 mmHg) in deficient 5.9% vs. sufficient: 5.2%; p=0.7988 | ● No correlation between Vitamin D with SBP and DBP ● Higher SBP with Vitamin D deficiency ● No differences in prevalence of HTN and DBP across Vitamin D status groups |
| Khadgawat, 2012- New Delhi, India [62] | Serum 25(OH)D: radioimmunoassay  Vitamin D status groups: Deficient:<50 nmol/L; Severe; Deficiency: <12.5 nmol/L; Moderate Deficiency: 12.5–25 nmol/L; Mild Deficiency :25–50 nmol/L SBP and DBP: average of 3 readings in a sitting position, after a 5 minutes rest, by a mercury sphygmomanometer in the right upper arm with an appropriate size cuff | BMI; Age; Gender; Pubertal stage | NS difference in SBP and DBP acroos vitamin D groups (serum 25(OH) D <10 ng/ml vs. ≥10 ng/ml)  SBP mean(SD): 118(12) vs. 124(12); p=0.06 DBP mean(SD): 78(7) vs. 80(9); p=0.55 | No differences in SBP and DBP across Vitamin D status groups |
| Hirschler, 2013- Buenos Aires, Argentine [54] | Serum 25(OH)D: radioimmunoassay kit  Vitamin D status groups: Deficient: <50 nmol/L SBP and DBP: NR | Age (adjustment only for SBP across quartiles) | Significant association between 25(OH) D and SBP across groups (All: 90(10); Insuficiency: 85(9); Deficiency 88(9); Severe deficiency:91(10); p<0.05) Significant association between 25(OH) D and DBP across groups(All: 56(9); Insuficiency: 52(8); Deficiency 55(9); Severe deficiency:58(9); p<0.05)  Significant Association between quartiles of serum 25(OH)D and SBP (Quartile I: 92(9); Quartile II: 90(10); Quartile III 89(8); Quartile IV: 86(9), p<0.05 | ● Higher SBP and DBP with Vitamin D deficiency ● Lower SBP with increasing quartiles of Vitamin D |
| Tomaino, 2015- Lima and Tumbes, Peru [97] | 25(OH)D: in duplicate, using the LIASON 25-OH vitamin D total assay Vitamin D status group: Deficient: <50 nmol/L; Non-deficient: ≥50 nmol/L SBP and DBP: median of 3 measurements, after 5-minute rest, using the right arm and in the seated position MAP: 1/3 SBP + 2/3 DBP | Overweight status; Age; Sex; Height; Seasonality; Personal smoking status; Second-hand smoke exposure; Monthly household income; Study site | Significantly higher BP in deficient vs. nondeficient subjects (DBP: 65.1(7.4) vs. 62.6(7.3); p<0.001; SBP: 111.7(10.4) vs. 109.8(11.4); p<0.01; MAP: 80.7(7.7) vs. 78.3(7.1); p<0.001)  25(OH)D deficiency associated with elevated DBP (point estimate = 1.09 mm Hg increase, 95% CI: 0.04 to 2.14; p= 0.04) 25(OH)D deficiency associated with elevated MAP (point estimate = 1.16 mm Hg increase, 95% CI: 0.10 to 2.22; p= 0.03)  SBP tended to increase with vitamin D deficiency (point estimate = 1.30 mm Hg increase, 95% CI:−0.13 to 2.72; p= 0.08)  SBP was found to demonstrate a U-shaped relationship with 25OHD, while DBP and MAP demonstrated inverse J-shaped relationships with serum 25OHD status | Higher MAP, SBP, DBP with Vitamin D deficiency |
| Bacha, 2019- Texas, USA [40] | Total 25(OH)D: electrochemiluminescence assay  SBP and DBP: average of 7 readings, taken 10 minutes apart, by an automated device | None | No statistical significant difference between the three tertiles of 25(OH)D and SBP [Tertile 1:106.7(1.9 ) Tertile 2: 107.2 (2.1) Tertile3:107.0 ( 1.8 )p=(NS)] No statistical significant difference between the three tertiles of 25(OH)D and DBP [Tertile 1:67.0 ( 1.2) Tertile 2: 65.6 (1.4 )Tertile364.2 (1.5):p=(NS)] | No differences in SBP and DBP across tertiles of Vitamin D |
| Banzato, 2014- Verona, Italy [41] | 25(OH)D: chemiluminescent method SBP and DBP: average of 3 readings, on the left arm over 30 minutes, in sitting position, using a mercury sphygmomanometer | None | NS association between tertiles of serum (OH)D and SBP[:All: 127(14.1);Tertile1: 136.6(13.5);Tertile 2:121.7(11.5); Tertile3: 122.7(13.4);p=0.06] NS association between tertiles of serum (OH)D and DBP[:All:67.6(13.9); Tertile1: 69.7(15.1);Tertile 2: 65(10.8)Tertile3:68.1(16); p=0.78] | No differences in SBP and DBP across tertiles of Vitamin D |
| Mellati, 2015-Iran [73] | Serum 25(OH)D: ELISA using immunodiagnostic system SBP and DBP: average of 3 readings in 10 min intervals after at least a 15-minute rest. For those with higher BP, the measurement was repeated on another day | None | Significant association between 25(OH) D and SBP (tertile I: 102.66(12.34); tertile II: 98.19(12.4), tertile III (96.33(12.05) p =0.012. Significant association between 25(OH) D and DBP (tertile I: 66.69(10.92); tertile II: 62.84(11.7), tertile III (59.83(9.78) p =0.014 25(OH)D level was negatively and significantly associated with SBP (r = –0.176, p = 0.002), DBP (r = –0.190, p = 0.001) | ● Lower SBP and DBP with increasing tertiles of Vitamin D ● Inverse correlation between vitamin D with SBP and DBP |
| Nam, 2012- South Korea [77] | Serum 25(OH)D: radioimmunoassay SBP and DBP: average of 2 measurements in 5-minute intervals, by standard mercury sphygmomanometer on the right arm | None | NS difference of SBP between tertiles of vitamin D SBP (mmHg): Tertile I: 104.70±0.80; Tertile II: 104.59±0.98; Tertile III: 104.14±0.81; p=0.868  Significantly higher DBP with lower serum 25(OH)D tertiles, specifically Tertile I vs. Tertile II: DBP (mmHg): Tertile I: 68.00±0.76; Tertile II: 65.47±0.82; Tertile III: 65.29±0.76; p=0.029 | ● No difference in SBP across tertiles of Vitamin D ● Lower DBP with increasing tertiles of Vitamin D |
| Ganji, 2011-USA [15] | Serum 25(OH)D: radioimmunoassay SBP and DBP: mercury sphygmomanometer (not detailed) | For SBP: Age; Sex; Race/ethnicity; BMI For DBP: Age; Sex | Significant inverse association between serum 25(OH)D and SBP (p for trend= 0.01) SBP was significantly higher in the lowest serum 25(OH)D tertile group than in the highest serum 25(OH)D tertile group [109.8(0.5) for Tertile 1 vs. 108.2(0.4) for Tertile 2; 108.4(0.4) for Tertile 3; p <0.0167] NS association between serum 25(OH)D and DBP (p for linear trend= 0.51) [59.9(0.4) for Tertile 1; vs. 60.0(0.5) for Tertile 2, 60.6(0.5) for Tertile 3; NS] | ● Lower SBP with increasing tertiles of Vitamin D ● No association between Vitamin D with DBP |
| Parikh, 2012- Augusta area, USA [86] | Plasma 25(OH)D: liquid chromatography tendem mass spectroscopy SBP and DBP: NR | Age; Gender; Ethnicity; Sexual maturation; Season; Physical activity; Percent body fat | Negative correlation between plasma 25(OH)D and SBP (r=-0.1; p=0.02) and DBP (r=-0.21; p<0.01) 25(OH)D levels significantly explained the variances in SBP (R2= 0.012, p= 0.04), and DBP (R2= 0.055, p<0.01) Significant association between tertiles of plasma 25(OH)D and SBP (mean(SE): 115(1) for Tertile I vs. 110(1) for Tertile II and 108(1) for Tertile III; p for trend <0.01) NS association between tertiles of plasma 25(OH)D and DBP (mean(SE): 60(1) for Tertile I vs. 60(0) for Tertile II and 60(1) for Tertile III; p for trend= 0.75) | ● Inverse correlation between Vitamin D with SBP and DBP ● Lower SBP with increasing tertiles of Vitamin D; No difference in DBP |
| Malyavskaya, 2017- Russia [108] | Serum 25 (OH)D: ELISA SBP and DBP: NR | None | No significant association among quartiles of 25 (OH) D and SBP (Quartile I: 109 (10) Quartile II: 108(10), Quartile III: 108(10), Quartile IV: 108(11) p=0.072; and p=0.051 for Q1 vs. Q4 No significant association among quartiles of 25 (OH) D and DBP (Quartile I: 69 (7) Quartile II: 67(6), Quartile III: 66(6), Quartile IV: 66(7) p=0.009; and p=0.028 for Q1 vs. Q4 Correlation analysis revealed no relationships between the level of 25 (OH) D and SBP (r = -0.15; p = 0.114)  Correlation analysis revealed negative relationships between the level of 25 (OH) D and DBP (r = -0.27; p = 0.005) | ● No differences in SBP and DBP across quartiles of Vitamin D, except for a higher DBP for Q 1 vs. 4 ● Inverse correlation between vitamin D with DBP only |
| Hirschler, 2012- Buenos Aires, Argentine [53] | Serum 25(OH)D: radioimmunoassay kit SBP and DBP: NR | Tanner stage | No significant association between quartiles and SBP of 25(OH) D (quartile I: 106(14); quartile II 96(11); quartile III 99 (11) quartile IV: 100(12)  No significant association between quartiles and DBP 25(OH) D (quartile I: 166(11); quartile II 60(9); quartile III 62(18) quartile IV: 62(11) Mean values of systolic blood pressure showed significant differences across quartile, but lost significance when adjusted for Tanner stages. | No differences in SBP and DBP across quartiles of Vitamin D |
| Lee, 2015- Seoul, South Korea [68] | 25(OH)D: 125I-labelled radioimmunoassay kits SBP and DBP: average of 2 readings in the right arm, within 5 minutes interval, by a standard mercury sphygmomanometer at rest | Age; Gender | Significant association between quartiles of 25(OH)D and SBP (Quartile I: 104.92 CI: 104.15-105.68; Quartile II: 103.89, CI: 103.11-104.66; Quartile III: 103.30 CI: 102.53-104.07; Quartile IV: 102.05 CI(101.32-102.78) p after adjustment for trend: 0.002 Significant association between quartiles of 25(OH)D and DBP (Quartile I: 66.45 CI: 65.78-67.12; Quartile II: 65.12, CI: 64.49-65.75; Quartile III: 63.76 CI: 63.13-64.39; Quartile IV: 62.4 CI(61.75-63.05) p<0.001, p for trend <0.001); p after adjustment for trend p<0.001   SBP (mean (95% CI)) 104.03±10.53 for Quartile I–III (<20.86) vs. 102.05±9.98 for Quartile IV (≥20.86); p<0.001 DBP (mean (95% CI)) 65.11±8.86 for Quartile I–III (<20.86) vs. 62.40±8.92 for Quartile IV (≥20.86); p<0.001 | Lower SBP and DBP with increasing quartiles of Vitamin D |
| Reis, 2009- USA [91] | Serum 25(OH)D: radioimmunoassay SBP and DBP: average of up to 4 measures by a mercury-gravity sphygmomanometer using appropriate arm cuff size, in seated position, after a 5-minute rest  High BP: SBP or DBP ≥90th p for age, sex, and height, or use of BP medications | Age; Gender; Ethnicity; BMI; Poverty-to-income ratio; Physical activity No adjustment for Vitamin D as outcome | Inverse association between 25(OH)D and SBP [mean (95%CI) SBP Q1: 109.7 (108.4, 111.0); Q2: 108.5 (107.5, 109.5); Q3: 108.1 (107.0, 109.2); Q4: 108.1 (107.3, 108.9); p= 0.02] NS association between 25(OH)D and DBP [mean (95%CI) DBP Q1: 61.7 (60.3, 63.1); Q2: 62.1 (60.8, 63.4); Q3: 61.2 (60.2, 62.2); Q4: 61.2 (60.0, 62.4); p= 0.082]  Inverse association between 25(OH)D and high BP [prevalence (95%CI) of high BP: Q1: 11.2 (7.6, 16.2); Q2: 6.7 (5.1, 8.7); Q3: 4.4 (3.1, 6.3); Q4: 3.7 (2.6, 5.3); p= 0.013; adjusted OR (95%CI): Q1: 2.36 (1.33, 4.19); Q2: 1.26 (0.65, 2.44); Q3: 1.04 (0.55, 1.97); vs. Q4; p= 0.046]  Lower mean (95% CI) 25(OH)D in nmol/l in High BP (SBP or DBP ≥90th percentile for age, sex, and height, or use of BP medications) group: 56.75(52.75, 60.75) vs. normal BP group: 62.5(60.5, 64.5); p=0.003 | ● Lower prevalence of high BP and SBP with increasing quartiles of Vitamin D; No difference in DBP ● Lower Vitamin D level among participants with high BP |
| Cabral, 2016- Porto, Portugal [42] | Serum 25(OH)D: chemiluminescence immunoassay  SBP and DBP: average of 2 readings separated by ≥ 5 minutes, after a 10 minute rest, by a mercury sphygmomanometer | BMI; Gender; Parental education; Physical activity; Season | NS diffrence among tertiles of 25(OH)D and SBP [Quartile1:113.8 (CI: 112.2–115.5) Quartile 2: 113.7 (CI: 111.6–115.8) Quartile 3:112.7 (CI:110.7–114.7) Quartile 4:111.3 (CI: 109.2–113.4) p=0.224] NS diffrence among tertiles of 25(OH)D and DBP[ Quartile1:67.4 (CI:66.2–68.7) Quartile 2:67.7 (CI:66.2–69.2) Quartile 3:66.8 (CI:65.4–68.4) Quartile 4:66.3 (CI:64.8–67.8)p=0.516]  NS difference in mean(SD) (nmol/l) vitamin D across high BP groups:  No BP: 41.5(14.22); BP: 40.7(14.42); p=0.550 | ● No differences in SBP and DBP across quartiles of Vitamin D ● No difference in Vitamin D level among normotensive and those with high BP |
| Nsiah-Kumi, 2012- Nebraska, USA [80] | 25(OH)D: radioimmunoassay  Vitamin D status groups: Deficient: <40 nmol/L; Insufficient: <75 nmol/L SBP and DBP: after a 5-minute rest, using an appropriately sized cuff | BMI p-for-age and-sex | Inverse association between 25(OH)D and SBP [Coefficient: -0.49; 95%CI: -0.75; -0.24; p=0.0001]  Inverse association between 25(OH)D and DBP [Coefficient: -0.49; 95%CI: -0.80;-0.19; p=0.016] | Inverse association between Vitamin D with SBP and DBP |
| Kelishadi, 2014-Iran [61] | Serum 25(OH)D: chemiluminescence immunoassay  SBP and DBP: average of two readings by a standardized mercury sphygmomanometer in a sitting position on the right arm with an appropriate cuff size | Age; Gender; Anthropometric measures | Significant weak inverse association of 25(OH)D level with SBP and DBP Model I: SBP R= 0.32 CI ( 0.05, 0.05) p= 0.94 DBP R=0.22 CI ( 0.04, 0.04) p=0.94 Model II: SBP CI (1.40, 2.02) p <0.001 DBP CI (0.64, 1.11) p<0.001 Model III: SBP: CI ( 0.62, 2.63) p<0.01 DBP: CI ( 0.88, 1.62) p <0.01 | Inverse association between Vitamin D with SBP and DBP |
| Ha, 2013- Suwon, South Korea [50] | Serum 25(OH)D: chemiluminescence immunoassay  SBP and DBP: average of 2 readings by an automated BP instrument | Age; Gender; Tanner stage; Body fatness; physical activity | NS betweenn quartiles of serum 25(OH)D and SBP ( Q1:105.3 (13.1) Q2:103.9 (9.9) Q3:104.6(9.4) Q4: 105.7(11.9)p=0.162 ) NS betweenn quartiles of serum 25(OH)D and DBP ( Q1:65.0(8.2) Q2: 65.1(7.1)Q3:62.9(6.1) Q4: 64.0(65)p= 0.23) NS association between SBP and 25(OH)D: unadjusted: gamma:-0.054, p=0.343; adjusted: gamma=-0.049,p=0.401) NS association between DBP and 25(OH)D: unadjusted: gamma:-0.182, p=0.001; adjusted: gamma=-0.089, p=0.127) | ● No differences in SBP and DBP across quartiles of Vitamin D ● No association between Vitamin D with SBP and DBP |
| Ghobadi, 2019- Shiraz, Iran [48] | Serum 25(OH)D: ELISA SBP and DBP: average of 2 readings, by mercury sphygmomanometer | Age; Gender; BMI; Physical activity | Negative significant association between Serum 25(OH)D and SBP (β = −0.02; CI: (−0.05, −0.004), and DBP (β = −0.02; CI: −0.05, −0.003) | Inverse association between Vitamin D with SBP and DBP |
| Hirschler, 2019- San Antonio de los Cobres and Chicoana, Argentine [55] | Serum 25(OH)D:radioimmunoassay kit SBP and DBP: average of 2 readings ,at a period of 1 to 2 minutes, by a mercury sphygmomanometer in a sitting position with the child’s right forearm horizontal on the table and cuffs sizes adjusted for differences in arm circumference and height  Hypertension: average of the values of SBP and /or DBP >=95th p based on age , sex and height percentiles | Age; Gender; z-BMI; Milk intake | CH showed an inverse correlation between vitamin D and SBP (r = −0.19, P < .05) Significant inverse associations between 25(OH)Dlevels and SBP (β = −.29; p < .01; R2 = .12) Significant inverse associations between 25(OH)Dlevels and DBP (β = −.18; p < .01; R2 = .06) | Inverse association between Vitamin D with SBP and DBP |
| Rafraf, 2014- Boukan, Iran [90] | Serum 25(OH)D: ELISA SBP and DBP: average of 2 measurements, at a 1-2 min interval, in the morning by a mercury sphygmomanometer with an adult cuff on the upper right arm, with the arm horizontally on a table, in the sitting position, after a 5-minute rest | BMI; Energy; Physical activity level | NS association between serum 25(OH)D and SBP (Standardized B: -0.184; p= 0.0107) NS association between serum 25(OH)D and DBP (Standardized B: 0.173; p=0.131) | No association between Vitamin D with SBP and DBP |
| Izadi, 2020- Tehran, Iran [57] | Serum 25(OH)D: ELISA SBP and DBP: standard mercury pressure gauge with dimensions suitable for children and standard medical devices in a sitting position after a 5 minute rest | Linear regression; Age; BMI; Gender; DBP; Triglycerides; HDL and Total Cholesterol | Significant inverse corrlation between serum 25(OH)D and SBP[r=-0·295; p=0·022] Significant inverse corrlation between serum 25(OH)D and DBP [r=-0.371; p=0·004] NS assocation between 25(OH)D and SBP (Standardized coefficient: 0.077; p=0.365) | No association between Vitamin D with SBP, in adjusted model |
| Hirschler, 2019- Argentine [56] | Serum 25(OH)D: radioimmunoassay SBP and DBP: average of 2 readings at a period of 1 to 2 minutes by a mercury sphygmomanometer in a sitting position, with the child’s right forearm horizontal on the table and cuffs sizes adjusted for differences in arm circumference and height MAP: (DBP*2 + SBP) / 3 | BMI; Age; Sex; Location; Triglycerides; Insulin; Glucose | NS association between vitamin D and mean arterial BP (non standaridzed beta: 0.14; SE: 0.15; p=0.36) NS association between vitamin D and SBP (data not shown) NS association between vitamin D and DBP (data not shown) | No association between Vitamin D with SBP, DBP and MAP |
| Moore, 2017-USA [75] | Serum 25(OH)D: LC/MS Vitamin D status group: Deficient: <50 nmol/L; Insufficient: 50–72.5 nmol/L; Sufficient :> 72.5 nmol/L SBP and DBP: average of 3 readings by the auscultatory method in a sitting position for 5 minutes Normal BP: SBP or DBP <90th p for age, sex and height; Pre-HTN: SBP: >= 90th to < 95th p for age, sex and height; HTN: >= 95th p for age, sex and height | Race/ethnicity; Sex; Age; Economic status; BMI-z score | Inverse correlatin between SBP and serum 25(OH)D (p<0.03), but not when also controlling for BMI (p=0.63)  Hypertension prevalence across vitamin D groups (p<0.0001) Normal BP: 97.6% in deficient, vs. 98% in insufficient and 98.9% in sufficient Pre-hypertension 1.8% in deficient, vs. 2.0% in insufficient, and 0.7% in sufficient Hypertension 0.7% in deficient, vs. 0% in insufficient, and 0.3 in sufficient  SBP mean(SE): 107.9(0.6) in deficient vs. 106(0.5) in insufficient, and 105.3(0.5) in sufficient (significant difference between deficient and sufficient) DBP mean(SE): 57.9(0.9) in deficient vs. 55.8(0.9) in insufficient, and 56.4(0.9) in sufficient [NS]  NS association between total 25(OH)D and SBP (standardized beta: -0.013; SE: 0.03; p=0.642) and DBP (standardized beta: 0.003; SE: 0.04; p=0.899) NS association between 25(OH)D3 and SBP (standardized beta: -0.014; SE: 0.03; p=0.630) and DBP (standardized beta: 0.008; SE: 0.04; p=0.780) NS association between 25(OH)D2 and SBP (standardized beta: 0.005; SE: 0.24; p=0.683) and DBP (standardized beta: -0.05; SE: 0.88; p=0.165) | ● No association between Vitamin D with SBP and DBP, in adjusted model ● Higher prevalence of HTN with poorer Vitamin D status |
| Petersen, 2015-Denmark [87] | Serum 25(OH)D (including both D2 and D3): automated chemiluminescent immunoassay SBP and DBP: average of 3 readings, after 10-minute rest, by an automated device, using two different cuff sizes | Sex; Age; Height; Ethnicity; Whole-blood EPA+ DHA; Entered puberty (yes/no); Parental education | NS association between 25(OH)D and SBP: (beta=-0.02, CI:−0·06, 0·00,p=0.12) [no adjustement] Significant association between 25(OH)D and DBP: (beta=-0.3, CI:−0·6,-0.00, p=0.02) Significant association between 25(OH)D and DBP after adjusting for fat mass index: (beta=-0.3, CI:−0·6, −0·0, p=0.04) Significant association between 25(OH)D and DBP after adjusting for moderate to vigorous physical activity: (beta=-0.3, CI:-0.5-0, p=0.06) | ● No association between Vitamin D with SBP ● Inverse association with DBP, in adjusted model |
| Williams, 2011-USA [100] | Serum 25(OH)D: radioimmunoassay SBP and DBP: average of up to 4 measurements, at rest | Age; Gender; Ethnicity; Poverty-income-ratio; Waist circumference; Sampling probability (via weights); Cluster effects | Inverse associations between z-scored 25(OH)D and SBP [Coefficient (95%CI): −0.068 (−0.118,−0.018); p= 0.009] For each greater nmol/l of 25(OH)D, SBP decreases on average by −0.032 (95% CI −0.055;−0.008) mm Hg NS association between z-scored 25(OH)D and DBP [Coefficient (95%CI): −0.046 (−0.105,0.014); p= 0.13] | Inverse association between Vitamin D and SBP only |
| De Moraes, 2014- European Countries [45] | Plasma 25(OH)D: immunoassay ELISA  SBP and DBP: lowest of 2 readings, taken 10 minutes apart, in sitting position, using an oscillometric monitor device | Contextual variables (seasonality; latitude of residence; school); Potential individual confounders (maternal education; age at menarche (in girls); BMI; waist circumference; physical activity; serum lipid concentrations); | No significant correlation between serum Vitamin D and systolic and diastolic BP in both girls and boys (p>0.05).  Girls:Adjusted beta for SBP= 0.01, 95% CI,-0.05-0.03 Girls adjusted beta for DBP= -0.01. 95%CI, -0.05-0.02 Boys:Adjusted beta for SBP= -0.05, 95% CI,-0.11-0.02 Boys:Adjusted beta for DBP= 0.01, 95% CI,-0.03-0.05 | No association between Vitamin D with SBP and DBP |
| Lee, 2014-Seoul, South Korea [14] | Serum 25(OH)D: chemiluminescent immunoassay  SBP and DBP: standard brachial cuff technique HTN: > 90th p for sex, height and age | BMI | NS assoiations between quartiles of 25(OH)D and SBP (Quartile I: 111.41 CI: 110-112.83; Quartile II: 110.16, CI: 108.82-111.5; Quartile III: 110.06 CI: 108.62-111.51; Quartile IV: 109.52 CI(108.11-110.93) p=0.30) NS assoiations between quartiles of 25(OH)D and DBP (Quartile I: 69.16 CI: 68.04-70.29; Quartile II: 68.77, CI: 67.73-69.8; Quartile III: 69.00 CI: 67.82-70.19; Quartile IV: 69.68 CI(68.56-0.80) p=0.70)  Prevalence of high BP: QI: 46.8%; Q2: 73.9%; Q3: 47.7%; Q4: 46.2%; p=0.98  Similar risk for high BP across quartiles of vitamin D Adjusted OR (95% CI): Q1: 0.91 (0.69-1.20); Q2: 0.94 (0.71-1.23); Q3: 0.91 (0.69-1.20); Q4: 1.00 (referent); p=0.90  NS difference in mean (SD) of 25(OH)D in nmol/l across BP groups: No BP: 46.67(12.97) vs. BP: 47.17(12.23); p=0.58 | ● No differences in prevalence of high BP, SBP and DBP across quartiles of Vitamin D ● No difference in Vitamin D level among normotensive and those with high BP |
| De piero Belmont, 2015-Spain [46] | Plasma 25(OH)D: immunochemiluminescence SBP and DBP: average of 3 reading, taken 5 minutes apart using the right arm, in sitting position HTN status: Normal BP: SBP or DBP ≤90th p; PreHTN: SBP or DBP ≥90th p and ≤95 thp; High BP: SBP or DBP ≥95th p | For the OR: Age; gender | Significant association among tertiles of serum 25(OH)D and SBP:(Tertile 1: 108.2±14.4; Tertile 2:106.2±13.9; Tertile 3: 103.8±13.2; P<0.05) Significant association among tertiles of serum 25(OH)D and DBP:(Tertile 1:65.2±10.4; Tertile 2:62.1±10.2; Tertile 3:63.8±11.0 ; P<0.05)  Hypertension (%) across tertiles (p<0.05): Normal: T1: 79.8; T2; 85.7; T3: 94.2  Prehypertension: T1: 6.7; T2: 5.1; T3: 1.0 Hypertension: T1: 13.5; T2: 9.2; T3: 4.9  SBP or DBP ≥90th percentile: OR (CI95%):  T1 Reference T2 0,59 (0,28-1,24) T3 0,50 (0,32-0,79) (p<0.01) | ● Lower SBP and DBP with increasing tertiles of Vitamin D ● Higher prevalence of HTN in the lowest tertile of Vitamin D compared with the highest tertile ● Lower odds of elevated SBP or DBP with increasing tertiles of vitamin D |
| Kim, 2018- South Korea [64] | Serum 25(OH)D: NR  Vitamin D status groups: Deficient: <50 nmol/L; Sufficient: ≥50 nmol/L SBP and DBP: average of 3 readings on the right arm after a 5-minute rest Elevated BP: SBP>=130mmHg; DBP>=85mmHg | Age; Gender; Household income; Residential area; Self perceived health status; Self-perceived stress status; family history of chronic disease; Sleep habits, and physical activity | SBP ≥130 mmHg or DBP ≥85 mmHg: 3.2% (SE: 1.0) in the sufficient group vs. 4.4% (SE: 0.6) in the sufficient group; p=0.369 The risk of elevated BP similar in the deficient and sufficient groups Model 1: OR=1.43 (0.69-2.94) p=0.333 Model 2: OR=0.78 (0.31-1.99) p=0.3604 Model 3: OR=0.72 (0.28-1.89) p=0.508 | Similar odds of elevated BP across Vitamin D status groups, in adjusted model |
| Nam, 2014- South Korea [78] | Serum 25(OH)D: radioimmunoassay Vitamin D status groups: Insufficient: ≤50 nmol/L; Sufficient: >50 nmol/L SBP and DBP: average of 2 measurements in 5-minute intervals, by standard mercury sphygmomanometer on the right arm High BP: SBP or DBP ≥90th p for age and sex, use of BP-lowering medication or a previous diagnosis of HTN | For the OR between high BP and 25(OH)D: Age; Gender; BMI; Regular physical exercise; Alcohol drinking; Use of multivitamin or mineral supplements | Significant negative correlation between serum 25(OH)D and SBP: r=-0.059; p=0.021 Significant negative correlation between serum 25(OH)D and DBP: r=-0.141; p=<0.0001  NS difference in SBP according to 25(OH)D levels: 107.1 (SE: 0.4) for insufficiency vs. 105.8 (SE: 0.7) for sufficiency; p=0.201 Significant higher DBP: 68.6 (SE: 0.4) for insufficiency vs. 66.4 (SE: 0.5) for sufficiency; p=0.002  High BP (fully adjusted model): OR for insufficiency vs. sufficiency: 1.07 (95%CI: 0.77-1.50; p=0.673) | ● Inverse correlation between Vitamin D with SBP and DBP ● No difference in SBP across Vitamin D status groups; higher DBP with Vitamin D insufficiency ● Similar odds of high BP across Vitamin D status groups, in adjusted model |
| Pacifico, 2011-Rome, Italy [85] | Serum 25(OH)D3: electrochemiluminescence immunoassay SBP and DBP: average of 2 measures, at the right arm in the supine position using an automated oscillatory system, after a 10-minute rest Elevated BP: SBP or DBP >= 90th p for age, gender and height | For correlation: age; gender; Tanner stage For regression:  Model 1: age; gender; Tanner stage Model 2: age; gender; Tanner stage; waist circumference Model 3: age; gender; Tanner stage; Standard deviation score-BMI | Inverse association between serum 25(OH)D3 tertiles and median SBP [median(IQR) for Tertile 1: 110(20); Tertile 2: 105(10); Tertile 3: 100(15); p<0.0001] Inverse association between serum 25(OH)D3 tertiles and median DBP [median(IQR) for Tertile 1: 70(10); Tertile 2: 65(10); Tertile 3: 62(10); p= 0.012]  Negative correlation between 25(OH)D3 and SBP [all cases: r=-0.183; p <0.0001; overweight/obese: r=-0.185; p<0.01; normal: r= -0.168; p <0.01] NS correlation between 25(OH)D3 and DBP [all cases: r= -0.102; overweight/obese: r= 0.080; normal: r= -0.085; p >0.05]  Higher odds of elevated BP with lower 25(OH)D Prevalence (95%CI): Tertile 1: 42.3( 34.4-50.2), Tertile 2: 37.6 (29.7-45.5), Tertile 3: 28.6 (21.2-36.0) Model 1: OR(95%CI) for Elevated BP: Tertile 1: 2.12 (1.28-3.51), Tertile 2: 1.60 (0.96-2.63) vs. Tertile 3 Model 2: OR(95%CI) for Elevated BP: Tertile 1: 1.70 (1.01-2.90), Tertile 2: 1.60 (0.94-2.73) vs. Tertile 3 Model 3: OR(95%CI) for Elevated BP: Tertile 1: 1.72 ( 1.02-2.92), Tertile 2: 1.18 (0.90-1.55) vs. Tertile 3 | ● Lower SBP and DBP with increasing tertiles of Vitamin D ● Negative correlation between Vitamin D with SBP only ● Lower odds of elevated BP with increasing tertiles of vitamin D, in adjusted model |
| Kumar, 2009-USA [65] | 25(OH)D: diasorin assay Vitamin D status groups: Deficient:< 37.5 nmol/L; Insufficient: 37.5-72.5 nmol/L SBP and DBP: average of 3 readings  HTN: SBP or DBP >95th p for the median height of each participant's age and gender or >140/90mmHg in those >= 17 years of age | Age; Gender; Race/ethnicity; Poverty income ratio; Obesity; Milk intake; Television and computer use; Vitamin D supplement use | Children and adolescents with 25(OH)D insufficiency had higher DBP than their counterparts with 25(OH)D sufficiency SBP: Δ insufficient vs. sufficient 0.78; 95%CI: 0.08-1.64; p=0.08; Δ deficient vs. sufficient 2.24; 95%CI: 0.98-3.50; p=0.001  DBP: Δ insufficient vs. sufficient 1.68; 95%CI: 0.20-3.16; p=0.03; Δ deficient vs. sufficient 1.60; 95%CI: 0.54-3.75; p=0.14  Children and adolescents with vitamin D insufficiency and deficiency were more likely to have hypertension than their counterparts with 25(OH)D sufficiency Prevalence of Hypertension % (%SE): 2.1%(0.6) in sufficient; 2.4%(0.3) in insufficient, and 5.5%(1.1) in deficient; p=0.02  Hypertension: OR insufficient vs. sufficient: 1.0; 95%CI: 0.50-2.00; p=0.96; OR deficient vs. sufficient 2.5; 95%CI: 1.00-5.90; p=0.04  Significant association between groups of 25(OH)D and SBP mean(SE): suficient 106.0(0.5), insuficient: 107.0(0.3), deficient: 110.0(0.6) p<0.001 Significant association between groups of 25(OH)D and DBP mean(SE): suficient 58.0(0.7), insuficient: 60.0(0.4), deficient: 61.0(0.6) p<0.014 | ● Higher odds of HTN with Vitamin D deficiency ● Higher SBP and DBP with poorer Vitamin D status |
| Milagres, 2017-Vicosa, Brazil [74] | Serum 25(OH)D: architect 25-OH Vitamin D assay  Vitamin D status group: Deficient: <50 nmol/L; Insufficient: ≥50 – <75 nmol/L; Sufficient: ≥75 nmol/L  SBP and DBP: average of 3 readings by an automatic inflation BP monitor in a sitting position with at least a 5-minute rest  Elevated BP: SBP or DBP ≥90th p for age, gender and height according to the VI Brazilian guidelines of HTN by the Brazilian Society of Cardiology | Age; Gender; Season; Ethnicity; PTH; Per capita income; Maternal schooling; Vitamin D intake; Sedentary behavior; Percentage of body fat (or other measures of adiposity) | NS association between vitamin D status and elevated BP: prevalence of elevated BP 48% in >=75 nmol/l vs. 52% in <75 nmol/l group; p=0·718 NS difference in prevalence of hypertension in children with concentration of vitamin D≥50 nmol/l (Poisson Regression=1.09; 95% CI: 0·41, 2·88; p=0.865) than in deficient children (PR= 1·79; 95% CI: 0·93, 3·43; p=0·082) | No difference in prevalence of elevated BP and HTN across Vitamin D status groups |
| Williams, 2014- Pennsylvania, USA [103] | Serum 25(OH)D: NR Vitamin D status groups: Deficient: <50 nmol/L; Insufficient: <75 nmol/L SBP and DBP: NR High BP: >95th p for age and sex | Age; Sex; Race; Location; Season; Insulin level; Hyperlipidemia; Total comorbidities | Higher SBP with hypovitaminosis D but not with vitamin D deficiency SBP: 119.7±15.6 in deficient vs. 117.0±16.4 in not-deficient; p=0.314 SBP: 119.7±16.8 in hypovitaminosis vs. 112.4±12.1 in sufficient; p=0.024  NS association between DBP and vitamin D status DBP: 65.7±9.9 in deficient vs. 64.8±8.5 in not-deficient; p=0.536 DBP: 65.7±8.9 in hypovitaminosis vs. 63.3±9.4 in sufficient; p=0.18  NS association between high BP and vitamin D status 41.2% in deficient vs. 58.8% in not-deficient; p=0.916 70.6% in hypovitaminosist vs. 29.4% in sufficient; p=0.533  Significant association between SBP (per 1 mm Hg increase) and hypovitaminosis D: OR: 1.04 (95%CI: 1.01-1.07); p=0.02 | Higher SBP with poorer Vitamin D status; no difference in prevalence of high BP and DBP |
| Xiao, 2020- China [105] | Plasma 25(OH)D: chemiluminescent immunoassay Vitamin D status group: Adequacy: <50 nmol/L; Inadequacy: >50 nmol/L SBP and DBP: average of last 2 reading out of 3, with 1–2 min intervals, after resting for at least 15 min, in a sitting position from the right arm using a suitable cuff size based on the arm circumference HTN: average SBP and/or DBP ≥95th sex, age and height-specific p for Chinese children and adolescents, or taking antihypertensive drugs | Age; Gender; Season of blood collection; Geographical location; Smoking; Drinking; Physical activity; Dietary vitamin D intake; BMI; Fat mass percentage; Muscle mass index | NS difference in prevalence of hypertension in boys: 21.2% in adequate vs. 24.3% in inadequate, and 22.7% in deficient; p=0.247 Significant higer prevalence of hypertension in girls: 15.4% in adequate, vs. 23.4% in inadequate, and 23.7% in deficient; p<0.001  Significant inverse correlation between 25(OH)D and SBP (all: r=-0.035; p=0.007; boys: r=0.022; p=0.227; girls: r=-0.107; p<0.001) NS correlation with DBP (all: -0.012; p=0.356; boys: r=0.038; p=0.035; girsl: r=-0.068; p<0.001)  Higher SBP with vitamin D deficiency or inadequacy than with vitamin D adequacy (p<0.05) in all subject and in girls. NS difference in boys Higher DBP with vitamin D inadequacy than with vitamin D adequacy (p<0.001) in all subjects. Higher DBP with decreased vitamin D levels in girls (p<0.001). NS difference in boys  Significantly higher odds of hypertension across vitamin D groups in all subjects and in girls; NS in boys All: OR in inadequacy vs. adequacy: 1.23 (95%CI: 1.01 to 1.50; Sig); OR in deficiency vs. adequacy: 1.08 (95%CI: 0.87 to 1.34; NS); p for trend=0.823 Girls: OR in inadequacy vs. adequacy: 1.54 (95%CI: 1.10 to 2.16; Sig); OR in deficiency vs. adequacy: 1.66 (95%CI: 1.16 to 2.39; Sig); p for trend=0.018 Boys: OR in inadequacy vs. adequacy: 1.13 (95%CI: 0.88 to 1.45; NS); OR in deficiency vs. adequacy: 0.89 (95%CI: 0.67 to 1.18; NS); p for trend=0.162  Significantly higher odds of hypertension in obese individuals with vitamin D insufficiency (joint effects of vitamin D insufficiency and obesity, including overweight, on BP): Normal weight + Insufficiency: OR: 1.19 (95%CI: 0.95 to 1.48) [NS] Obesity + Adequacy: OR 2.49 (95%CI: 1.73 to 3.60; Sig) in comparison with Normal weight + Sufficiency Obesity + Insufficiency: OR 2.23 (95%CI: 1.70 to 2.93; Sig) in comparison with Normal weight + Sufficiency | Higher odds of HTN with Vitamin D inadequacy, in the total sample and in girls only |
| Muhairi, 2013- Al Ain, UAE [76] | Serum 25(OH)D: radioimmunoassay SBP and DBP: average of 2 readings by a standard mercury sphygmomanometer after a 5-minute rest in a sitting position with appropriate cuff size | None | NS difference in mean (95%CI) of 25(OH)D in nmol/l among those who had elevated blood pressure 57.9(51.16-64.64) vs. those with normal blood pressure 59.15(56.16-62.15) nmol/l; p=0.342 | No difference in Vitamin D level among normotensive and those with high BP |
| Oliveira, 2014-Juiz de Fora, Brazil [83] | Serum 25(OH)D: radioimmunoassay SBP and DBP: average of the second and third measurements, at 5-min intervals, with the right arm at the same level as the heart, by an equipment validated against mercury sphygmomanometers according to the international validation protocol, using an appropriate cuff size, in a sitting position Elevated BP: based on parameters of the Brazilian HTN Society taking into account gender, age and height | None | Lower serum 25(OH)D in nmol/l in adolescents with hypertension: median, IQR: 24.9(13.2) in BP<90th percentile vs. 21.3(10.4) in BP≥90th percentile; p=0.017 | Lower Vitamin D level among hypertensive participants |
| Yousefichaijan, 2019- Arak, Iran [106] | Serum 25(OH)D: NR Vitamin D status group: Deficient: <50 nmol/L SBP and DBP: digital monitor citizen Hypertensive status: Normal: BP< 90th p for age, gender and height; Pre-HTN: BP: 90-95th p; Stage 1 HTN: BP: 95-99th p + 5 mmHg; Stage II HTN: BP: >99th pe + 5 mmHg | None | NS association between 25(OHD)D and systolic HTN [mean(SD) 25(OH)D in nmol/l in normal SBP group: 34.22(11.01); pre-HTN group: 32.75(20.89); stage I HTN group: 23.34(NR); p: 0.624] NS association between 25(OHD)D and diastolic HTN [mean 25(OH)D in normal DBP group: 34.27(11.13); pre-HTN group: 28.78(12.18); p: 0.409] | No difference in Vitamin D level across BP status groups |
| Teixeira, 2018- Rio de Janeiro, Brazil [96] | Serum 25(OH)D: HPLC Vitamin D status group: Deficient: ≤50 nmol/L; Insufficient: >50-<75 nmol/L; Adequate: 75-247 nmol/L SBP and DBP: average of 2 measures taken 1 min apart, by oscillometric technique semi-automatic digital arm device, after a 5-minute rest HTN: according to the VI Brazilian Guidelines for HTN in adolescents | None | NS association between vitamin D status and hypertension [prevalence of hypertension: 67% in the inadequate vitamin D status group vs. 40% (data from figure) and 60% (data from text) in the adequate vitamin D status group (NS)] | No difference in prevalence of HTN across Vitamin D status groups |
| **Retrospective** | | | | |
| Aypak, 2014- Ankara- Turkey [39] | 25(OH)D: imunochemiluminescent assay SBP and DBP: NR | None | NS correlation between serum 25(OH)D and SBP[ r= -0.044, p= 0.586] NS correlation between serum 25(OH)D and DBP[ r= 0.1, p=0.212] | No correlation between Vitamin D with SBP and DBP |
| Gul, 2017- Tokat- Turkey [49] | Serum 25(OH)D: chemiluminescence immunoassay  Vitamin D status groups: Deficient: <37.5 nmol/L; Insufficient: 37.5-72.5 nmol/L; Sufficient: ≥75 nmol/L SBP and DBP: NR HTN: BP>= 95th p for age, sex and height | None | NS differences were observed between 25(OH) D groups with respect to hypertension frequency (22.8% in deficient group; 15% in insufficient group and 0% in Sufficient group; p = 0.074)  NS differences were observed between 25(OH) D groups with respect to mean SBP and DBP: SBP: mean(SD): 116.73(14.32) in deficient vs. 114.83(12.61) in insufficient, and 112.2(13.69) in sufficient; p=0.357 DBP (mean(SD): 73.88(11.28) in deficient vs. 73.64(9.65) in insufficient, and 72(9.36) in sufficient; p=0.859  NS correlations of 25(OH) D and SBP (r = -0.099 and p = 0.082) and DBP (r = -0.065 and p = 0.256)  Significantly lower mean(SD) 25(OH)D in nmol/l across hypertension groups: No Hypertension: 36.19(20.56); Hypertension: 29.75(13.67); p=0.004 | ● No differences in HTN frequency, SBP and DBP according to Vitamin D status groups ● No correlation between Vitamin D with SBP and DBP ● Lower Vitamin D level among hypertensive participants |
| Smotkin-Tangorra, 2007- New York, USA [95] | 25(OH)D: NR Vitamin D status groups: Insufficient: <50 nmol/L SBP: 1 measure in seated position | None | SBP significantly higher in the insufficiency group [mean(SD): 117(17)] than in sufficiency group [mean(SD): 111(17)] | Higher SBP with Vitamin D insufficiency |
| Kao, 2015- Australia [16] | Serum 25(OH)D: electrochemiluminescent immunoassay or direct chemiluminescence competitive immunoassay SBP and DBP: manual sphygmomanometer in a seated position with appropriate cuff size | BMI; Age; Gender; Season | Significant association among quintiles and SBP (Quintile I: 123.7 (2.3), Quintile: II 122.4 (2.4); Quintile III: 119.5(2.1), Quintile IV: 119.5(2.9), Quintile V: 114.1 (2.8) p=0.03 Significant association among quintiles and DBP (Quintile I: 70.8(1.6), Quintile: II 70.3 (1.3); Quintile III: 69.2(1.4), Quintile IV: 68.4(1.4), Quintile V: 64.8 (1.8) p=0.009; Quintile I : OR 4 (1.34-11.91) p=0.01; Quintile II : OR 3.81 (1.31-11.08) p=0.01 | ● Lower SBP and DBP with increasing quintiles of Vitamin D ● Higher odds of elevated BP with lower quintiles of Vitamin D |
| **Case-control** | | | | |
| Liang, 2018- China [70] | Serum 25(OH)D: HPLC SBP and DBP: average of 3 readings in the sit-down position by electronic sphygmomanometer using an appropriately sized BP cuff placed on the right arm | BMI | Serum 25(OH)D in children with hypertension was lower than that in the control group (38.22(12.00) vs. 43.28(12.33) nmol/L, P=0.02) , whereas the difference of 25(OH)D level between the two groups was reduced after adjusted BMI (39.10(12.00) vs. 42.44(12.33) nmol/l; P=0.19) and the deficiency rate of 25(OH)D was boundary significant (P=0.06)serum 25(OH)D in children with hypertension | Lower Vitamin D level among participants with HTN; NS after adjustment |
| **Interventional (baseline assessment)** | | | | |
| Al Daghri, 2016- Riyadh, Saudi Arabia [109] | Serum 25(OH)D: COBAS e-411 automated analyzer SBP and DBP: average of 2 readings, at rest | None | No significant correlation between serum 25(OH)D and SBP[r= 0.1; p.0.05] Significant inverse correlation between serum 25(OH)D and DBP[r=-0.23; p<0.05] | Inverse correlation between vitamin D with DBP only |
| Khayyatzadeh, 2018- Iran [63] | Serum 25(OH)D: electrochemiluminescence  Vitamin D status groups: Deficient: <50 nmol/L; Insufficient :50-74.9 nmol/L; and Sufficient: > 75 nmol/L SBP and DBP: standard procedure | None | NS association between serum 25(OH)D and SBP (All: 96.4 (14.2); Def: 96.6(14.2); Insuf: 98.3(14.3); Suf: 98.8(11.2) p=0.49 NS association between serum 25(OH)D and DBP ( All: 62.3(13.4);Def: 62.5(13.05); Insuf: 64.5(12.8); Suf: 66.05(10.4) p=0.17 | No differences in SBP and DBP across Vitamin D status groups |
| Ohlund, 2020- Umea and Malmo, Sweden [82] | Serum 25(OH)D2 and 25(OH)D3: MS on an API 4000 LC/MS/MS system (AB Sciex) SBP and DBP: using an automated oscillometric sphygmomanometer in Umea, and an automatic BP monitor in Malmo | Gender; Skin color; Study site; Mothers’ education | Significant negative association between serum 25(OH)D and SBP (adjusted β = −0.194; 95% CI: −0.153, −0.013) Significant negative association between serum 25(OH)D and DBP (adjusted β = −0.187; 95% CI: −0.150, −0.011) | Inverse association between Vitamin D with SBP and DBP, in adjusted model |
| Smith, 2018- United Kingdom [94] | Serum 25(OH)D: liquid chromatography-tandem mass spectrometry SBP and DBP: average of 3 measurements, 1-min apart, using an automatic BP monitor, in upright position with the arm supported | Sex; Age; BMI z-score; Tanner stage; Physical activity | NS association between serum 25(OH)D and SBP (Adjusted B: 0.01; 95%CI: −0.02, 0.04; p=0.48) NS association between serum 25(OH)D and DBP (Adjusted B: 0.00; 95%CI: −0.04, 0.03; p=0.88) | No association between Vitamin D with SBP and DBP |
| **Longitudinal (baseline assessment)** | | | | |
| Kwon, 2015- South Korea [67] | Serum 25(OH) D: radioimmunoassay SBP and DBP: average of 2 readings, 5 minutes apart, by an automatic device with the correct cuff size and the arm properly supported | Age; Sex; BMI z-score; Birth order; Fruit/fruit juice intake; Maternal educational level | NS association between quartiles of serum 25(OH) D and SBP (quartile I: 104.4(1.26); quartile II: 100.83(1.23), quartile 3: 101.03(1.21), quartile 4: 101.64(1.24) p=0.46 NS association between quartiles of serum 25(OH) D and DBP (quartile I: 60.1(0.98); quartile II: 58.8(0.86), quartile 3: 58.43(0.95), quartile 4: 59.54(0.97) p=0.60 NS association between serum 25(OH) D and SBP in all models:Model 1: β=-0.16, p=0.20; Model 2: β=-0.08, p=0.54 NS association between serum 25(OH) D and DBP in all models:Model 1: β=-0.06, p=0.53; Model 2: β=0.01, p=0.95 | No association between Vitamin D with SBP and DBP, in adjusted model |
| Williams, 2012- Avon, Southwest England [101] | 25(OH)D: HPLC Season-adjusted 25(OH)D3 Total 25(OH)D: sum of 25(OH)D2 and unadjusted 25(OH)D3 SBP and DBP: mean of 2 measurements, using a Vital Signs monitor, at rest, with the arm supported at chest level | Age; Gender; Ethnicity; Socioeconomic position; Waist circumference; PTH; Circulating calcium and phosphate level | NS associations between season-adjusted 25(OH)D2 and SBP and DBP SBP (mm Hg): Mean difference: 0.13; 95%CI: 0.20, 0.46; p=0.43 DBP (mm Hg): Mean difference: 0.01; 95%CI: 0.26, 0.24; p=0.92 Mean differences: a unit change in outcome per doubling of 25(OH)D2 | No association between Vitamin D with SBP and DBP, in adjusted model |
| **Longitudinal (baseline assessment) and cross-sectional** | | | | |
| Nandi-Munshi, 2017- USA [79] NHANES (2001-2006): Cross-sectional SNAS: Prospective cohort (baseline assessment) | NHANES:  Serum 25(OH)D: radioimmunoassay SBP and DBP: average of up to 3 measures, at rest, using a mercury sphygmomanometer BP: normal: SBP and DBP <90th p; pre-HTN: SBP or DBP ≥90th - <95th p; HTN: SBP or DBP ≥95th p  SNAS: Serum 25(OH)D: chemiluminescence immunoassay based on a linkage between specific vitamin D antibody-coated magnetic particles and an isoluminol derivative SBP and DBP: average of 3 measures, using a mercury sphygmomanometer HTN: BP >90 p for age, sex, and height, or use of antihypertensive drugs | None | NHANES: NS association between 25(OH)D and BP categories [mean(95%CI in nmol) 25(OH)D: 61.75(60-63.5) in normal BP group vs. 61.75(57.5-66) in pre-hypertension group and 55.75(50-61.5) in hypertension group; p=0.098]  SNAS: NS association between 25(OH)D in nmol/l and BP categories [mean(SD) 25(OH)D: 60(34) in normal BP group vs. 58(33.25) in hypertension group; p=0.88] | No difference in Vitamin D level among normotensive and those with HTN |
